# Structural Comparative Modeling of Multi-Domain F508del CFTR

**DOI:** 10.3390/biom12030471

**Published:** 2022-03-18

**Authors:** Eli Fritz McDonald, Hope Woods, Shannon T. Smith, Minsoo Kim, Clara T. Schoeder, Lars Plate, Jens Meiler

**Affiliations:** 1 Department of Chemistry, Vanderbilt University, Nashville, TN 37235, USA; eli.f.mcdonald@vanderbilt.edu (E.F.M.); clara.schoeder@medizin.uni-leipzig.de (C.T.S.); lars.plate@vanderbilt.edu (L.P.); 2 Center for Structural Biology, Vanderbilt University, Nashville, TN 37235, USA; hope.woods@vanderbilt.edu (H.W.); shannon.t.smith.1@vanderbilt.edu (S.T.S.); 3 Program in Chemical and Physical Biology, Vanderbilt University, Nashville, TN 37235, USA; min.soo.kim.1@vanderbilt.edu; 4 Leipzig Medical School, Leipzig University, 04109 Leipzig, Germany; 5 Department of Biological Sciences, Vanderbilt University, Nashville, TN 37235, USA; 6 Department of Pharmacology, Vanderbilt University, Nashville, TN 37235, USA; 7 Institute for Drug Discovery, Leipzig University, 04109 Leipzig, Germany

**Keywords:** cystic fibrosis, comparative modeling, computational protein modeling, protein folding disease, pharmacological chaperones, VX-809, structure-based drug discovery

## Abstract

Cystic fibrosis (CF) is a rare genetic disease caused by mutations in the cystic fibrosis transmembrane conductance regulator (CFTR), an epithelial anion channel expressed in several vital organs. Absence of functional CFTR results in imbalanced osmotic equilibrium and subsequent mucus build up in the lungs-which increases the risk of infection and eventually causes death. CFTR is an ATP-binding cassette (ABC) transporter family protein composed of two transmembrane domains (TMDs), two nucleotide binding domains (NBDs), and an unstructured regulatory domain. The most prevalent patient mutation is the deletion of F508 (F508del), making F508del CFTR the primary target for current FDA approved CF therapies. However, no experimental multi-domain F508del CFTR structure has been determined and few studies have modeled F508del using multi-domain WT CFTR structures. Here, we used cryo-EM density data and Rosetta comparative modeling (RosettaCM) to compare a F508del model with published experimental data on CFTR NBD1 thermodynamics. We then apply this modeling method to generate multi-domain WT and F508del CFTR structural models. These models demonstrate the destabilizing effects of F508del on NBD1 and the NBD1/TMD interface in both the inactive and active conformation of CFTR. Furthermore, we modeled F508del/R1070W and F508del bound to the CFTR corrector VX-809. Our models reveal the stabilizing effects of VX-809 on multi-domain models of F508del CFTR and pave the way for rational design of additional drugs that target F508del CFTR for treatment of CF.

## 1. Introduction

**Figure 1:**
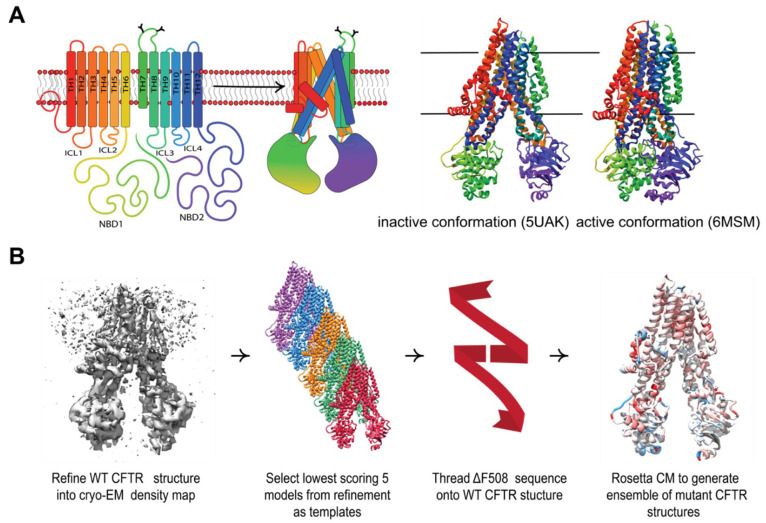
Comparative modeling captures multi-domain CFTR thermodynamics. (**A**) The complex topology of CFTR involves interdomain contacts formed during the folding process that include intercellular loops (ICLs) interfacing with the cytosolic NDBs. The inactive PBD ID 5UAK [2] (left) and active PBD ID 6MSM [6] (right). (**B**) Our workflow for generating ensembles of F508del models in this study (see Methods).

Cystic fibrosis (CF) is caused by mutations in the cAMP-regulated, phosphorylation gated anion channel cystic fibrosis transmembrane conductance regulator (CFTR) [1]. CFTR is a member of the ATP-binding cassette type C (ABCC) transporter family composed of two nucleotide binding domains (NBDs), two transmembrane domains (TMDs), and a flexible regulatory domain [2]. CFTR undergoes a complex domain-domain assembly (Figure 1A) during biogenesis and folding. Deletion of phenylalanine 508 in NBD1 (F508del) is observed in 70% of patient alleles [3] and thus represents the most common cause of CF and target for drug development. F508del destabilizes CFTR resulting in premature degradation and gating malfunction [4]. The CF patient phenotype lacks CFTR mediated anion transport at the epithelial apical plasma membrane in the lung epithelia, hindering osmotic regulation, and preventing cilia at the tissue/air interface from recycling mucus. Mucus build-up leads to poor lung function, is prone to infection, and ultimately leads to death [5].

At present, CF treatment includes channel gating potentiation and CFTR folding correction through small molecules called potentiators and correctors, respectively [7,8,9,10]. However, these compounds may interfere with birth control [11], cause testicular pain [12], and results in mental health side effects such as depression and psychotic symptoms [13]. Understanding the atomic level mechanisms of CFTR correctors can facilitate computational design of improved CF therapeutics with fewer side effects. Recent studies elucidated the structural basis of potentiator compounds [14,15]. Furthermore, cryo-EM [16] and computational modeling [17] revealed the binding site of FDA approved corrector VX-809 to WT CFTR. However, VX-809 binding to its primary target in the clinic, F508del CFTR, remains poorly understood.

VX-809 stabilizes F508del at the NBD1/TMD interface [18,19] and importantly, F508del requires both NBD1 and NBD1/TMD interface correction to function [20,21,22,23]. Thus, understanding the structural effects of F508del on NBD1 and the NBD1/TMD interface with atomic resolution offers a basis for rational, structure-based drug design. Previous studies have used NBD1 crystal structures to simulate F508del and understand the atomic effects on this single domain [24,25,26]. However, despite the importance of the NBD1/TMD interface for F508del correction, few studies leveraged recently published human multi-domain CFTR structures to model F508del CFTR [27,28]. These studies used homology modeling or zebrafish CFTR to generate starting models for molecular dynamics, but no study has leveraged the published human cryo-EM CFTR structures to model F508del [27,28] and no study has yet modeled F508del bound to VX-809. Furthermore, no experimental structures of F508del CFTR have been determined to date.

Here, we used Rosetta to model WT and F508del CFTR. We first refined an ensemble of WT CFTR Rosetta models into the cryo-EM density [29] and then used the lowest scoring refined models as templates for Rosetta comparative modeling (RosettaCM) [30] to model F508del (Figure 1B). We tested the optimal template number against thermodynamic data published on CFTR second site suppressor (SSS) NBD1 mutants [20,21,31]. Next, multi-domain WT and F508del CFTR structures including TMD1, NBD1, TMD2, and NBD2 were modeled using RosettaCM. We discussed our results in the context of abundant biochemical information about F508del CFTR folding [32,33,34]. In addition to unfolded conformations, F508del CFTR can adopt a near-native state that may sample similar conformational space to WT, and we attempted to model these F508del near-native states. Nevertheless, our models successfully captured F508del CFTR thermodynamic destabilization consistent with folding defects in NBD1 and at the NBD1/TMD interface. Next, we modeled F508del/R1070W CFTR and demonstrated its ability to locally stabilize the NBD1/TMD interface. Finally, working under the hypothesis that VX-809 binds fully translated F508del, we modeled F508del CFTR bound to VX-809 using RosettaCM and showed that drug binding decreased the total energy of the active state, but also stabilized the local region in the inactive state. To our knowledge, this study presents the first attempt to model the multi-domain F508del CFTR protein in silico bound to a corrector compound and used methods compatible with computer-aided drug design in the Rosetta Software Suite, a first step towards rational drug design for CF treatment.

## 2. Materials and Methods

### 2.1. Protein Structural Data Preparation

The dephosphorylated (inactive) human CFTR cryo-EM structure was downloaded from the PDB (5UAK [2], resolution 3.9 Å, determined residues 5–402, 439–645, 845–883, 909–1172, 1207–1436). The residues from a poorly determined helix between the TMDs were removed from the 5UAK PDB file manually. We also downloaded the phosphorylated (active) human CFTR cryo-EM structure from the PDB (6MSM [6], resolution 3.2 Å, determined residues 1–409, 435–637, 845–889, 900–1173, 1202–1451) and removed lipids and an unresolved helix near the lasso motif manually, ATP was kept in both binding sites. 6MSM contains the stabilizing mutation E1371Q and we used the MutateResidue mover in Rosetta to revert this back to E in our model. Finally, we also downloaded the raw cryo-EM density maps for 5UAK and 6MSM from the PDB.

For our testing set, we prepared a NBD1 structure from 5UAK by truncating the published model at residue Y385 through the determined portion of NBD1 to residue M645 (note this excludes the RI region from 403–438). We modeled ATP into the degenerate site by aligning 5UAK and 6MSM and copying the MG and ATP coordinates from 6MSM into the NBD1 structure. This resulted in an NBD1 structure including ATP bound at the degenerate site for our testing set.

### 2.2. Cryo-EM Refinement

We refined the published coordinates into the raw cryo-EM density maps using a previously established method in Rosetta [29] (see Protocol Capture Step 1). This approach requires the published structure coordinates and the published cryo-EM density map (both available on the PDB) as well as a set of refinement parameters (Figure S1, Supplementary Materials). We optimized refinement parameters including the weight put on the cryo-EM density, the length of fragment insertion and the distance of fragment insertion to increase ensemble diversity. We evaluated ensemble diversity by calculating the structural alpha carbon (Cα) root mean squared deviations (RMSD) from the published model for each refinement ensemble and assumed a greater Cα RMSD distribution indicated a more diverse ensemble. We optimized the sampling weight put on the cryo-EM data (denswt), the root mean squared distance (RMS) for peptide fragment insertion, and the length of the peptide fragments (Figure S1, Supplementary Materials).

### 2.3. Optimization of Cryo-EM Refinement Parameters

We optimized the user specified parameters required for Rosetta cryo-EM refinement [29]. First, to avoid overfitting, we optimized the weight put on the experimental density data (denswt) in the refinement score function. We generated 100 structures at density weight values between 20 and 50 at 5-point intervals. Next, we plotted these density weight values versus the difference between the Fourier shell correlation (FSC) and 4% of the per-residue energy for the ensemble (FSC–0.04*per-residue energy). The maximum difference indicates the optimal density weight. We chose a density weight value of 30 as this maximizes the (FSC–0.04*per-residue energy) value for most structures (Figure S1A,B, Supplementary Materials). Second, to maximize structural diversity, we optimized the length of peptide fragment insertion. The refinement protocol builds possible models by breaking sequences of determined residues into peptide fragments of an odd number length (e.g., 5, 7, 9, 11, or 13) [29]. Increasing the insertion length increases model diversity (28). We increased the fragment insertion length from seven to thirteen, generated 100 models for each, and plotted the Cα RMSD of each model in the refinement from the published model versus the model score for all four CFTR structures. Indeed, increasing the fragment insertion length from seven to thirteen generated overall lower scoring models with a greater Cα RMSD distribution for both the inactive (PDB ID 5UAK) and active (PDB ID 6MSM) states (Figure S1C,D, Supplementary Materials). Thus, we chose 13 for our fragment length. Third, we optimized the root mean squared (rms) distance between the inserted peptide fragments by varying this value from 1.5 to 2.5 Å in intervals of 0.25 and generating 100 models per interval. We plotted the Cα RMSD of the most poorly determined domain-NBD1-vs. the model score. We plotted NBD1 as this domain will likely have the greatest distribution in structural diversity from refinement. We chose a rms value of 1.75, as this value increases the NBD1 Cα RMSD (Figure S1E,F, Supplementary Materials). Increasing the rms value beyond 1.75 offered no improvement in NBD1 Cα RMSD (data not shown for clarity).

### 2.4. In Silico Mutagenesis

We made point mutations in CFTR structures using the MutateResidue mover in Rosetta. For the phosphorylated model, 6MSM, we mutated E1371Q back to the naturally occurring glutamine residue. The low structural resolution makes side chains difficult to distinguish in regions of NBD1, near F508. Hence to generate deletion mutations, we removed F508 from the active and inactive state CFTR fasta files respectively and threaded the sequence onto the active and inactive state models. For our testing NBD1 structure, we again deleted F508 from the NBD1 fasta sequence (residue 385–402 and 439–645) and threaded the new sequence onto the NBD1 structure. We mutated all second site suppressor mutations (F494N, F494N/Q637R, V510D, I539T, and G550E/R553Q/R555K) in NBD1constructs prepared for our testing set using the MutateResidue mover in Rosetta.

### 2.5. Rosetta Comparative Modeling

To model CFTR variants, we used RosettCM, a homology modeling approach [30]. We perform CM with static templates derived from the cryo-EM density, not the cryo-EM density itself. Published CFTR structures contain undetermined loops and intrinsically disordered regions including the RI region, the RD, the glycosylation site, and the loop linking TMD2 to NBD2. We generated fasta files containing only the determined residues in 5UAK and 6MSM (Table S2, Supplementary Materials). To generate F508del templates, we manually removed F508 from the fasta files and threaded the ΔF sequence onto the five WT models (see in silico mutagenesis section). As a control we used the WT sequence and the original WT templates and performed the same modeling protocol. Additionally, we mutated R1070W into the F508del templates and substituted the W manually to the fasta sequence to model F508del/R1070W CFTR.

We performed multiple template hybridization with the Hybridize mover in Rosetta guided by the RosettaMembrane energy function [35,36]. We imposed membrane specific Rosetta energy terms within the theoretical membrane bilayer by predicting the transmembrane helix regions with OCTOPUS [37]. We set all template weights to 1.0. For fragment insertion, we used three and nine peptide long fragments with short and long fragment insertion weights set to 1.0. We optimized side chain positions by simulated annealing, also known as rotamer packing in Rosetta. We refined final models using one round of FastRelax mover (e.g., repeat = 1) in Rosetta which performs steepest gradient decent minimization in Cartesian coordinate space without constraints.

### 2.6. Calculation of Protein Stability Metrics

We evaluated protein thermodynamic stability metrics for WT, F508del, and F508del/R1070W CFTR. We calculated the alpha carbon (Cα) root mean squared deviation (RMSD) for whole structures as well as on a per-residue basis with respect to a reference model (either the published model or a low scoring model in the ensemble). For our per-residue Cα RMSD calculations we first aligned individual domains to account for any shifts in the domains relative to each other as we were interested only in local fluctuations. We assumed local Cα RMSD as a surrogate for protein flexibility. Furthermore, we calculated the residue interaction potential energy, which provides the potential energy in REU between every pair of contacting side chain in the structure. Finally, we calculated Rosetta scores for our NBD1 testing with second site suppressor mutations with ref2015 and calculated Rosetta scores using the membrane scoring function [35,36].

### 2.7. Docking and Parameterizing VX-809

An automated docking tool, Autodock Vina [38] version 1.1.2, Scripps Research Institute (La Jolla, CA, USA), was employed to dock parameterized VX-809 on the CFTR active cryo-EM structure (PDB-ID: 6MSM). Top 20 binding modes in the energy range of 100 kcal/mol were computed. Grid parameters: center (x, y, z) = (165.453, 168.303, 149.074), size (x, y, z) = (20, 20, 20). To attain the full atom parameters for VX-809, we created low energy 3-dimensional ligand conformations in Corina given 2D representation exported from Chem draw. We then checked the BCL-based basic chemistry for appropriate bond lengths, atom types, etc. Next, we generated ligand conformers using BCL ConformerGenerator [39] for 8000 iterations and clustered based on distance between individual conformers. We then made Rosetta-readable parameters file for ligand docking and comparative modeling. This takes the conformer SDF and assigns partial charges and points to the conformer file. This also outputs centroid and torsional parameter files, which are used in the comparative modeling with CFTR. We then performed full atom docking in Rosetta using RosettaLigand [40].

## 3. Results

### 3.1. Refining CFTR Models into Available Cryo-EM Density Data

To model F508del, we sought to effectively sample CFTR conformational space in silico and generate a biophysically realistic set of template structures using the cryo-EM density maps–the only experimentally verified models of multi-domain human CFTR. These CFTR structures are well determined in the TMDs (2.4 Å) but poorly determined in the NBDs (4.8–6Å) resulting in low average resolutions ranging from 3.2 to 3.9 Å [2,6]. This motivated us to refine the WT structures into the cryo-EM density maps according to a previously established approach [29]. Refinement generated a diverse set of models that are inherently accessible in the cryo-EM density map [29]. For RosettaCM [41], we chose a subset of refined models to use as templates.

**Figure 2:**
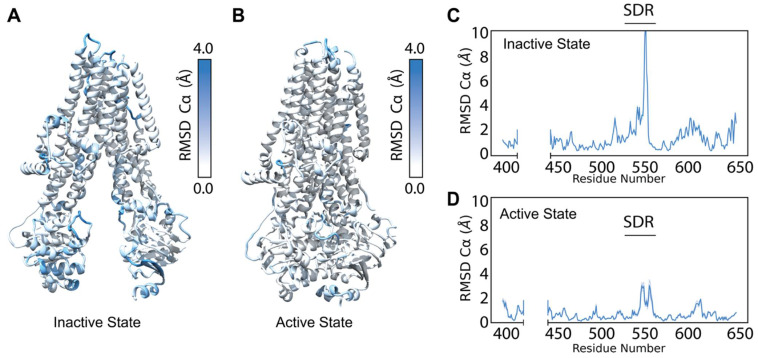
Refinement into the cryo-EM density generates a diverse ensemble of structures. (**A**) Average Cα RMSD of the best scoring (lowest 10% by potential energy function) 5UAK cryo-EM refinement models mapped onto 5UAK. (**B**) Average Cα RMSD of the best scoring (lowest 10% by potential energy function 6MSM cryo-EM refinement models mapped onto 6MSM. (**C**) The average NBD1 Cα RMSD of the best scoring 100 5UAK refinement models. The blue shading represents a 95% confidence interval, and the large Cα RMSD demonstrates high structural diversity in the SDR (residues 526–547). (**D**) The average NBD1 RMSD of the best scoring 100 6MSM refinement models. The blue shading represents a 95% confidence interval.

After optimizing refinement parameters for structural diversity (Figure S1, Supplementary Materials, see Methods), we refined 2000 models into the cryo-EM density maps [29] for the human dephosphorylated/inactive conformation (PDB ID 5UAK) [2] and the human phosphorylated/active conformation (PDB ID 6MSM) [6]. We note that 6MSM resolved no pore region but was determined in the phosphorylated and active channel state, thus we refer to 6MSM derived models as the “active” conformation here [42]. To evaluate the WT CFTR ensemble diversity, we calculated the Cα per-residue root mean squared deviation (RMSD) for each conformation from the respective published structure. Next, we mapped the average Cα per-residue RMSD onto the respective CFTR model from 0–4 Å to demonstrate visually which regions of CFTR show higher RMSD and are thus interpreted as inherently more flexible (Figure 2A,B). The ensemble demonstrated no substantial change in flexibility after 1000 models had been generated. Thus, we chose to stop generating models after model 2000 assuming a good sampling of the available conformational space.

Overall, the poorly determined NBDs showed greater structural diversity than the TMDs, as measured by RMSD from the published model (Figure 2A,B). Likewise, in the inactive state, NBDs showed greater structural diversity than the active state (Figure 2A,B). This likely resulted from the dimerized NBDs in the active state, which increased stability and lead to higher resolution cryo-EM data. Thus, the inactive conformation offered a greater sampling in the refinement ensemble than the active state. Refinement resulted in an ensemble of CFTR models with diverse conformations of loop regions such as extracellular loops and NBD1 loops.

We plotted the Cα per-residue RMSD for NBD1 in the active and inactive state to compare which sub-domains and regions demonstrated greater structural diversity between the conformations (Figure 2C,D and Figure S2A,B, Supplementary Materials). Notably, the structurally diverse region (SDR), residues 527–547, showed substantial increased Cα RMSD in both conformations, consistent with the known flexibility of the region (Figure 2C,D) [43]. These data suggest our cryo-EM refinement ensemble successfully captured the conformational flexibility of CFTR consistent with previous experimental studies.

### 3.2. Testing F508del Modeling with CFTR NBD1 Second Site Suppressor Mutations

We sought to accurately model F508del CFTR by leveraging the models generated during cryo-EM refinement. Although it remains unclear if F508del CFTR samples the same conformational space as the WT cryo-EM maps, F508del CFTR trafficks and gates properly at low temperatures [44], suggesting F508del adopts near-native conformations. Our method should model the near-native conformations of F508del. Given a novel sequence, RosettaCM samples the conformational space of homologous models called templates [30]. Instead of a novel sequence and homologous models, we used the F508del sequence and WT cryo-EM refinement models with the lowest potential energy scores as templates (see Methods).

We restricted our simulations to NBD1 (residues 385–402 and 435–644) because experimental CFTR thermodynamic data are only available for NBD1 [20,21,31] (Table S1, Supplementary Materials). Considering all residues with determined coordinates from the inactive (5UAK) and active (6MSM) state NBD1 (residues 385–402 and 439–637), these regions superimposed well with an RMSD of 2.23 Å, lower than the published resolution of either structure [2,6] (Figure S3A, Supplementary Materials). Thus, we chose to test only the inactive state NBD1 as the lower resolution offers more conformational sampling and the two structures are similar.

We tested the number of cryo-EM templates for sufficient sampling in RosettaCM. On one hand, too few templates offer little conformational information, on the other hand, too many templates bias the cryo-EM density. We deem sufficient sampling the optimal number of templates that correlate best with experimental data. To test the template number, we simulated NBD1 CFTR with second site suppressor (SSS) mutations using 3, 4, 5, 7, and 9 templates (Figure S3, Supplementary Materials). The resulting Rosetta scores were correlated with experimental ΔTm and ΔΔG data. Specifically, we used NBD1 experimental values with respect to WT NBD1 in each study to account for distinct experimental conditions between studies (Table S1, Supplementary Materials).

**Figure 3:**
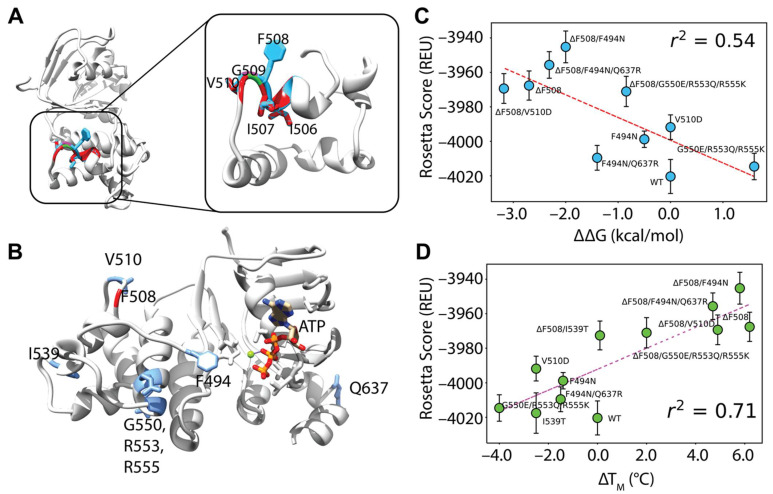
Comparative modeling of F508del NBD1 using five templates correlates well with experimental data. (**A**) An overlay of WT and F508del CFTR NBD1 structures at the H3/H4 loop. WT is depicted in blue and F508del is depicted in red with just the α-helical subdomain shown for clarity. Deletion of F508 leaves surrounding residues I506, I507 and V510 relatively unaltered. (**B**) The model for testing included NBD1 bound to ATP. Residues mutated in second site suppressor mutations are shown in blue including F494N, V510, I539, G550, R553, R555, and Q637. (**C**) Testing correlation between Rosetta score given in REU and ΔΔG values from the literature (Table S1, Supplementary Materials). Error bars represent standard error of the mean. Error in experimental data likely lies lower than ±1–2 kcal/mol. R squared represents Pearson correlation coefficient. (**D**) Testing correlation between Rosetta score given in REU and ΔTM values from the literature (Table S2, Supplementary Materials). Error bars represent standard error of the mean. Error in experimental data likely ranges with ±1–2 C. R squared represents Pearson correlation coefficient.

First, we generated F508del models by threading the F508del fasta sequence onto the WT model. Deletion of F508 left a gap and failed to perturb the NBD1 structure but prematurely terminated helix 3 (H3) causing the loop connecting H3 and H4 to shift (Figure 3A). This is consistent with the loop shift observed experimentally in the F508del NBD1 crystal structure [45]. Likewise, I506 and I507 side chains remained in their location when compared to WT (Figure 3A). Furthermore, G509 was pulled closer to H3 but fails to form a backbone hydrogen bond with I507. The V510 side chain moved only slightly in our models, tightening the H3-H4 loop (Figure 3A). This contrasts with the F508del NBD1 crystal structure where V510 changes from an “inward facing” to a solvent exposed orientation and suggests a limitation to threading the F508del sequence onto WT refined models (Figure S4A, Supplementary Materials) [45,46]. However, threading the F508del sequence was chosen instead of using previously published F508del NBD1 crystal structures because this method is more broadly applicable to rare CFTR mutants which have no published experimental structural data.

We included ATP at the degenerate binding site because NBD1 is known to fold with ATP as a scaffold at this site [47] (Figure 3B). Further, we simulated WT and F508del CFTR NBD1 with stabilizing mutations called SSS mutations. We included NBD1 SSS mutations F494N, F494N/Q637R, V510D, I539T, and G550E/R553Q/R555K (Figure 3B) because experimental CFTR thermodynamic data are available for these SSS [20,21,31].

We generated 1000 models for each SSS mutation combination and took the average Rosetta score of the lowest scoring 5% of models. Next, the Rosetta score versus the ΔT_m_ and ΔΔG were plotted for each SSS mutation and we calculated the Pearson correlation coefficient between the Rosetta scores and the experimental values. Of note, the experimental ΔT_m_ values correlated with the experimental ΔΔG values with an r^2^ of 0.78 which we subsequently assumed represents a good correlation (Figure S3B, Supplementary Materials).

We determined that using 3, 4, 5, 7, and 9 templates resulted in a Rosetta score-ΔT_M_ Pearson correlation coefficient of 0.22, 0.45, 0.71, 0.59, and 0.22 respectively and a Rosetta score-ΔΔG correlation of 0.14, 0.27, 0.54, 0.57, and 0.25 respectively (Figure 3C,D, and Figure S3C–G, Supplementary Materials). Thus, five templates offered the best correlation (Figure 3C,D).

### 3.3. F508del Destabilizes Inactive and Active State of Human CFTR

CFTR is unique among ABC transporters family proteins to function as a phosphorylation gated anion channel. F508del CFTR gates inefficiently and requires potentiators such as VX-770 to stabilize the active conformation [15]. Given the clinical importance of CFTR channel gating, we modeled F508del in both the inactive and active conformations.

**Figure 4:**
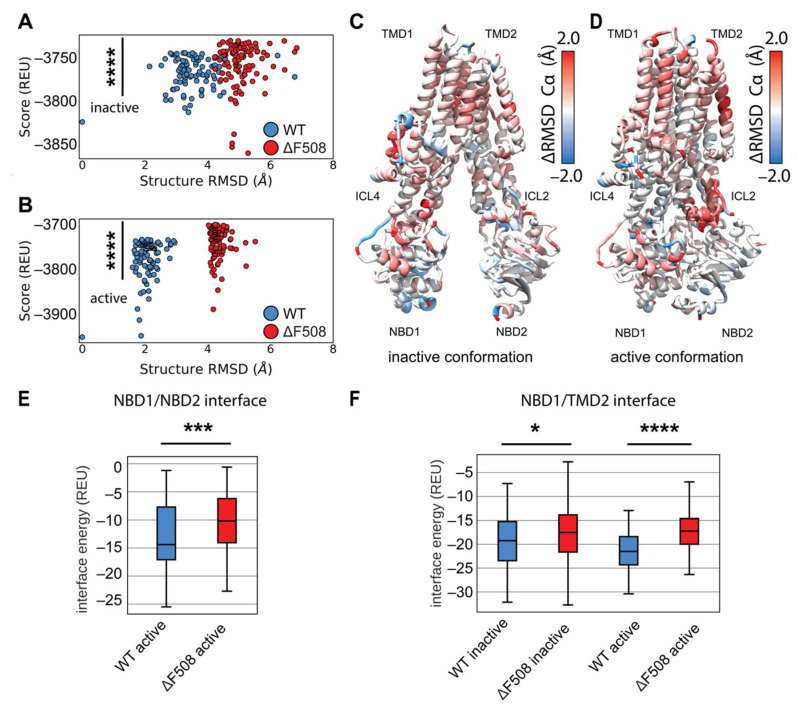
Comparative modeling of multi-domain F508del CFTR shows thermodynamic instability and lose of interaction energy at key domain-domain interfaces. (**A**) Cα RMSD vs. score plot of the lowest scoring 100 inactive conformation models from ensembles of WT (blue) and F508del (red) CFTR. RMSD is calculated relative to the lowest scoring WT model. Score is shown in REU. Statistical significance was calculated with a Mann–Whitney U test and all *p*-values are depicted by * < 0.05, *** < 0.001, and **** < 0.0001. (**B**) Cα RMSD vs. score plot of the lowest scoring 100 active conformation models from ensembles of WT (blue) and F508del (red) CFTR. (**C**) Average residue Cα RMSD of the lowest scoring 100 inactive state WT models subtracted from the Cα RMSD of the lowest scoring 100 inactive state F508del models mapped on 5UAK. Here, red represents region where the RMSD was higher in F508del than WT, and blue represents regions where the RMSD was lower. (**D**) Average residue Cα RMSD of the lowest scoring 100 inactive state WT models subtracted from the Cα RMSD of the lowest scoring 100 inactive state F508del models mapped on 6MSM. (**E**) Quantification of the residue–residue interactions at the NBD1/NBD2 interface across the lowest scoring 100 models. Only the active state is considered as the inactive state lack the NBD dimer and hence there are no residue interactions to measure. (**F**) Quantification of the residue-residue interactions at the NBD1/TMD2 interface across the lowest scoring 100 models. The box limits represent the upper and lower quartile with a line at the median, the whiskers represent 1.5 times the interquartile range, statistical significance was calculated with a Mann–Whitney U test.

We generated 2000 structure ensembles of WT and F508del CFTR using RosettaCM for both the inactive (PDB ID 5UAK) [2] and active (PDB ID 6MSM) [6] CFTR conformations. We examined the lowest scoring 100 models in terms of Rosetta score (Rosetta Energy Units or REU), which represented the best scoring 5% of the models generated. We plotted structural Cα RMSD (relative to the lowest scoring WT model) vs. Rosetta score to determine global structural changes among the mutant models (Figure 4A,B). WT and F508del models showed distinct structural shifts as measured by RMSD (Figure 4A,B). Furthermore, the F508del models showed statistically significant energy increases compared to WT in terms of REU (Figure 4A,B). These data suggest our models captured F508del thermodynamic instability in both conformations.

We sought to determine where F508del confers thermodynamic instability to the CFTR structure. First, we compared our multi-domain CM models to published crystal structures (Supplemental Figure S4B,C). Our models deviate little from the single domain crystal structures except in the α-helical subdomain, residues ~500–550, particularly the H3-H4 loop and SDR–indicating this region may change in multi-domain F508del (Figure S4B,C, Supplementary Materials). We used the residue RMSD from the lowest scoring model in each ensemble as a surrogate for structural flexibility associated with thermodynamic instability. Hence, we compared the flexibility of the lowest scoring 100 models in each WT and F508del ensembles in both conformations (Figure S5, Supplementary Materials). We subtracted the WT residue RMSD from the F508del residue RMSD and mapped the difference onto the published inactive and active conformations (Figure 4C,D). Here, red represents regions where F508del increased flexibility and destabilized the structure. By this metric, F508del demonstrated higher flexibility for both conformations in the α-helical subdomain (residues 500–540), specifically in helix 4B following F508 (Figure 4C,D and Figure S5, Supplementary Materials). The intercellular loops (ICLs) also demonstrated higher RMSD in F508del, particularly ICL4 in the inactive state (Figure 4C and Supplemental Figure S5A) and ICL2 in the active state (Figure 4D and Figure S5B, Supplementary Materials). These data suggest our multi-domain F508del reproduces the known destabilizing effects particularly in NBD1 and the NBD1/ICL interface.

Finally, multi-domain CFTR models allowed us to examine the energetic changes at the domain-domain interfaces. We calculated the residue interaction energy between all residues in the structures. We plotted the interaction energies between domains for the best scoring 100 models in terms of REU (Figure S6, Supplementary Materials). Next, WT and F508del were compared by summing the interaction energy across the interface and plotting the distribution of sums as boxplots (Figure S7A, Supplementary Materials). F508del significantly reduces the strength of the residue interactions between NBD1 and NBD2 in the active state (Figure 4E), consistent with the notion that F508del drives NBD2 unfolding in vivo [33]. Furthermore, F508del significantly reduces the strength of residue interactions between NBD1 and TMD2 (Figure 4F), which has long been suggested to be the predominant folding defect of F508del [20,21]. Finally, F508del significantly increases the interface energy between TMD1/NBD2 and TMD1/TMD2 in the active state as well (Figure S7B, Supplementary Materials). These data suggest our models captured the thermodynamic instability of the NBD1/NBD2 dimer interaction and the NBD1/TMD2 interface expected for the mutant consistent with experimentally verified destabilizing effects on these interfaces [20,21].

### 3.4. Modeling F508del/R1070W in Multi-Domain CFTR Lowers Interactions Energy at the NBD1/TMD2 Interface

**Figure 5:**
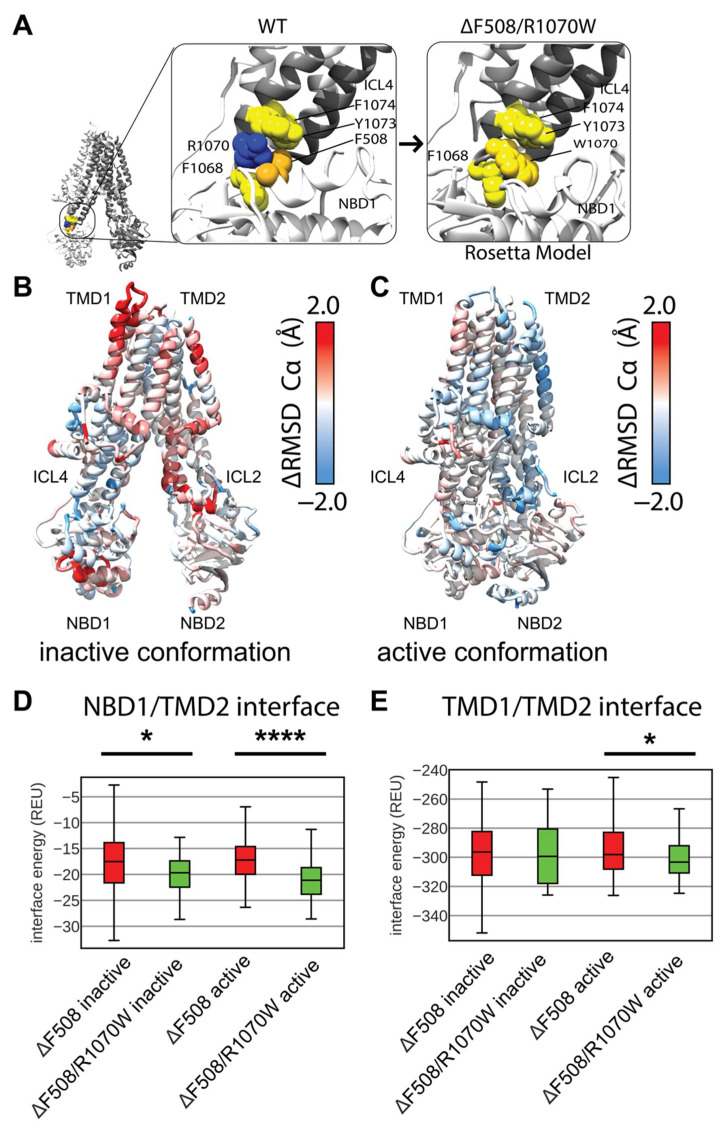
R1070W stabilizes the NBD1/TMD2 interface. (**A**) F508 in 5UAK CFTR (gold spheres) contacts an aromatic pocket in ICL4 formed by F1068, Y1073, and F1074 (yellow spheres). This aromatic pocket is filled with R1070 (blue) is mutated to a tryptophan (left). (**B**) Average residue Cα RMSD of the lowest scoring 100 inactive state F508del /R1070W models subtracted from the Cα RMSD of the lowest scoring 100 inactive state F508del models mapped on 5UAK. Here, red represents region where the RMSD was higher in F508del/R1070W than F508del alone, and blue represents regions where the RMSD was lower and hence stabilized by R1070W. (**C**) Average residue Cα RMSD of the lowest scoring 100 inactive state F508del /R1070W models subtracted from the Cα RMSD of the lowest scoring 100 inactive state F508del models mapped on 6MSM. (**D**) Quantification of the residue-residue interactions at the NBD1/TMD2 interface across the lowest scoring 100 models. The box limits represent the upper and lower quartile with a line at the median, the whiskers represent 1.5 times the interquartile range, statistical significance was calculated with a Mann–Whitney U test and *p*-values are depicted by * < 0.05, and **** < 0.0001. (**E**) Quantification of the residue-residue interactions at the TMD1/TMD2 interface across the lowest scoring 100 models. R1070W likely stabilize TMD2 enough to reduce the interaction energy between the TMDs in the active conformation.

Deletion of F508 leaves the aromatic pocket in ICL4 formed by F1068, Y1073, and F1074 empty, but the CFTR SSS mutation R1070W introduces a tryptophan into this pocket rescuing folding (Figure 5A) [20,21,48]. Interestingly, F508del/R1070W resisted correction by VX-809 indicating the SSS mutant and drug function via a similar mechanism stabilizing the NBD1/TMD interface [18]. Thus, R1070W represents a clinically relevant SSS to study in the context of multi-domain CFTR structure. To further evaluate our multi-domain CFTR modeling approach, we simulated F508del/R1070W and examined its effect relative to WT and F508del CFTR.

We again generated 2000 structure ensembles of F508del/R1070W and examined the lowest scoring 100 models in terms of Rosetta score. We determined the ensemble structural shift and thermodynamic changes conferred by R1070W by plotting structural RMSD vs. Rosetta score. F508del/R1070W shifted the ensemble structure very little from F508del models as measured by RMSD, and increased the energy (Figure S8, Supplementary Materials). These data suggest R1070W may globally destabilize the F508del structure in our models by increasing the overall score.

Thus, we sought to determine if R1070W conferred any local structural changes to the protein. We mapped the difference in residue RMSD of the lowest scoring 100 F508del/R1070W models vs. the lowest scoring 100 F508del models onto the published inactive and active conformations (Figure 5B,C). Here, red represents regions unstable in F508del/R1070W and blue represents regions unstable in F508del. By this metric, R1070W stabilized F508del more effectively in the active state compared to the inactive state (Figure 5C). R1070W reduced flexibility in the NBD1 α-helical subdomain of both conformations (Figure S9A,B, Supplementary Materials). These data indicate our multi-domain F508del/R1070W models reduced local F508del thermodynamic fluctuations, particularly in the active state, consistent with R1070W stabilizing effects on F508del CFTR [20,21]. However, R1070W increased global energy of the multi-domain models, which contrasts with experimental data and suggests limitations of our conformational sampling method using the cryo-EM density and/or our Rosetta scoring method for membrane proteins.

R1070W stabilizes the NBD1/TMD interface in vitro [20,21]. To study this effect in our models, we calculated the residue interactions energies for each of the best scoring 100 structures. Consistent with experiment, R1070W reduces the NBD1/TMD2 interface energy compared F508del alone in the inactive and active conformations (Figure 5D). Furthermore, R1070W reduced the TMD1/TMD2 interface energy compared to F508del in the active conformation (Figure 5E), showing a reduction towards WT levels of interface energy (Figure S10, Supplementary Materials). R1070W also reduced the energy of the TMD1/NBD2 interface (Figure S10, Supplementary Materials). These data suggest R1070W, despite having higher total potential energy in terms of Rosetta scores, primarily conferred local stability in the TMD interfaces.

### 3.5. Modeling Multi-Domain F508del CFTR Bound to VX-809

**Figure 6:**
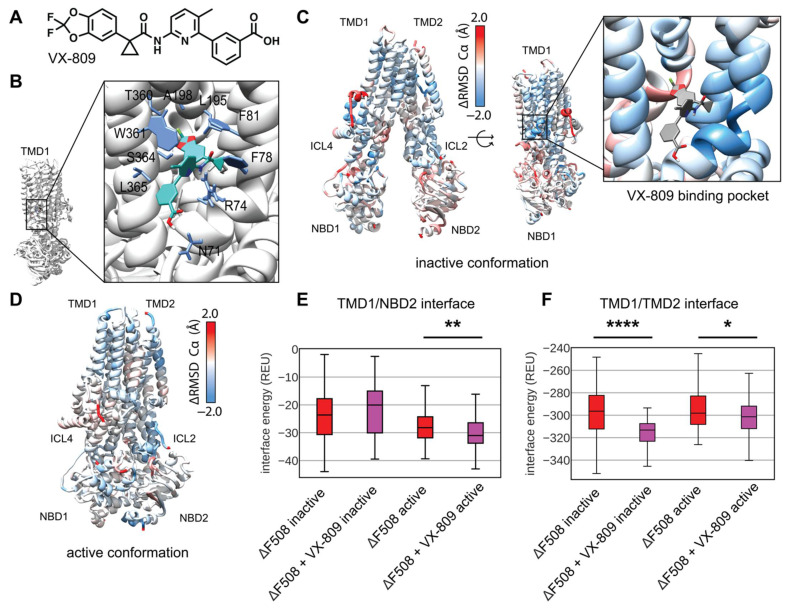
Comparative modeling of VX-809 bound to TMD1 F508del reveals local stability changes including the TMD1 domain-domain interfaces. (**A**) VX-809 chemical structure. (**B**) VX-809 docked to 6MSM CFTR structure in a putative binding site recently published by two parallel studies [16,17]. Interactions with important residues are shown in blue, VX-809 is shown in green with colored hetero-atoms. (**C**) Average residue Cα RMSD of the lowest scoring 100 inactive state F508del+VX-809 models subtracted from the Cα RMSD of the lowest scoring 100 inactive state F508del models mapped on 5UAK. Here, red represents region where the RMSD was higher in F508del +VX-809 than F508del alone, and blue represents regions where the RMSD was lower hence the structure was stabilized by VX-809. The inset shows the RMSD of the region surrounding VX-809 demonstrates reduced RMSD. (**D**) Average residue Cα RMSD of the lowest scoring 100 inactive state F508del+VX-809 models subtracted from the Cα RMSD of the lowest scoring 100 inactive state F508del models mapped on 6MSM. (**E**) Quantification of the residue-residue interactions at the TMD1/NBD2 interface across the lowest scoring 100 models. The box limits represent the upper and lower quartile with a line at the median, the whiskers represent 1.5 times the interquartile range, statistical significance was calculated with a Mann–Whitney U test and *p*-values are depicted by * < 0.05, ** < 0.01, and **** < 0.0001. (**F**) Quantification of the residue-residue interactions at the TMD1/TMD2 interface across the lowest scoring 100 models.

Current CF drug treatment uses small molecules called correctors to stabilize F508del CFTR including the FDA approved compound VX-809 (Figure 6A). Recently, two studies converged on a putative binding site for VX-809 to TMD1 of the WT CFTR protein [16,17]. Several previous biochemical studies alternatively suggested VX-809 and similar Type-I correctors may work by binding to NBD1 or the NBD1/ICL4 interface and subsequently stabilizing the NBD1/TMD2 interface through allostery [18,22,49,50], and VX-809 may have more than one binding site. While VX-809 may bind and stabilize partially folded CFTR intermediates [51], several studies suggest VX-809 binds and stabilizes trafficking competent F508del CFTR which may exist in near-native conformations [18,52]. Working under the hypothesis that VX-809 binds and stabilizes fully translated near-native F508del CFTR, we sought to model VX-809 in our F508del comparative models using the TMD1 binding site to determine the energetic changes VX-809 confers to F508del CFTR. To our knowledge, this represents the first attempt to model VX-809 bound to its primary pharmacological target, F508del CFTR.

First, we docked VX-809 in Autodock to the active conformation to get an initial binding pose of non-hydrogen atoms and determine the central coordinates of the molecule. Next, we used these central coordinates to dock a full atom model into binding pocket of active conformation in Rosetta and observed the lowest scoring 10 models in terms of interface energy score. We chose a binding pose that closely resembled published cryo-EM binding site which includes interactions with W361, T360, A198, L195, F81, F78, R74, and N71 (Figure 6B) [16]. Next, the full atom docked coordinates were copied into each F508del template in the inactive and active state for RosettaCM.

We modeled and analyzed F508del CFTR bound to VX-809 as described above. VX-809 increased the overall energy in the inactive state (Figure S11A, Supplementary Materials), however, VX-809 reduced the overall energy in the active state (Supplemental Figure S11B). To look at local fluctuations changes we mapped the difference in Ca RMSD between VX-809 bound and unbound F508del CFTR onto the inactive state model (Figure 6C and Figure S12A, Supplementary Materials). Here, blue represents areas where VX-809 reduced flexibility and stabilized F508del CFTR. Notably, VX-809 reduce the RMSD in the binding pocket in the inactive state (Figure 6C). We also mapped the RMSD difference onto the active state revealing VX-809 reduced flexibility in NBD1 and ICL2 (Figure 6D and Figure S12B, Supplementary Materials).

VX-809 allosterically stabilizes the NBD1/TMD interface in vitro [18,23]. We calculated the residue interaction energy between the domain/domain interfaces in the presence of VX-809. We found that VX-809 increased the energy of the NBD1/NBD2 interface in our model (Figure S13, Supplementary Materials); however, VX-809 reduced the TMD1/NBD2 interface energy in the active state (Figure 6E). Furthermore, VX-809 reduced the TMD1/TMD2 interface energy in both conformations (Figure 6F).

Interestingly, VX-809 had little effect on F508del/R1070W CFTR. We simulated F508del/R1070W with VX-809 bound in the same manner described above. These data show VX-809 fails to stabilize F508del in the presence of R1070W (Figure S14A–D, Supplementary Materials) consistent with the notion that the drug and stabilizing mutant have overlapping mechanisms stabilizing the NBD1/TMD2 interface [18]. Importantly, VX-809 had no effect on the F508del/R1070W NBD1/TMD2 interface interaction energy (Figure S14E, Supplementary Materials).

Thus, including VX-809 in our F508del CFTR comparative models showed a reduction in overall energy in the active state. The inactive state was destabilized by contrast. We speculate the discrepancy in conformational stability stems from accessibility of the VX-809 binding site in the active vs. inactive state. Recently published bound structures show an open binding pocked in the inactive conformation [16] compared to the originally published inactive models [2] where the binding site remains occluded. Thus, our method of placing VX-809 in this occluded pocket may have caused steric hinderance that increased the energy of our models. Nevertheless, VX-809 also reduced the local RMSD in the inactive state around the binding site and reduced the energy in the TMD1/TMD2 interface.

## 4. Discussion

In conclusion, we used Rosetta to develop multi-domain models of F508del CFTR, the primary drug target for CF. There remains a need for methods that can efficiently model large proteins, particularly important drug targets. We combined cryo-EM refinement with RosettaCM to model F508del and compare it to WT modeling as a control. Our models captured the thermodynamic instability of F508del in both the inactive and active conformations, particularly interactions at the NBD1/TMD2 interface. These models provide a basis for computer-based drug design of CFTR correctors to target and stabilize F508del CFTR or rarer CFTR variants. Specifically, mutant CFTR models may be generated with RosettaCM and emerging CFTR correctors and potentiators may be modeled and analyzed with RosettaLigand [53]. In addition, rare CFTR mutant comparative models may be used for virtual high throughput screening [54] to search for novel CFTR correctors that stabilize currently untreatable mutations.

Previous studies have used computer models to understand F508del effects on the structure of NBD1. However, few studies have attempted to understand F508del effects on multi-domain CFTR [27,28], despite the recent publications of multi-domain WT CFTR cryo-EM structures. No study to date has modeled multi-domain F508del CFTR bound to corrector VX-809. F508del folding defects stem from structural defects in both NBD1 and the NBD1/TMD interface, and F508del correction requires fixing both defects [20,21]. Hence, it is imperative to develop multi-domain models of CFTR to gain insight into the atomic level interactions underlying these structural defects.

Here, we used comparative modeling in Rosetta (RosettaCM) as it offers the computational speed required for virtual drug screening. Our method can quickly generate an ensemble of CFTR variant models that preserve hydrogen bond networks from mutants while maintaining the surface binding pockets of WT CFTR from the cryo-EM density. This offers the advantage of small molecule docking into comparative models [53], a computationally efficient technique pioneered by our group to allow ligand docking with conformational sampling. Combining RosettaCM with Biochemical Library (BCL) small molecule design [55] will allow CFTR mutant specific computational drug design and personalized medicine for CF.

We used recently published cryo-EM models of full length CFTR—with an undetermined regulatory domain—to refine an ensemble of WT models in the inactive and active conformations. The lowest scoring models from refinement were used as templates for RosettaCM by threading the F508del sequence onto the structure. We tested the number of templates required to capture F508del thermodynamic instability by simulating 1000 models of WT and F508del NBD1 with and without SSS mutations with known ΔΔG and ΔT_M_ values in the literature. Using five cryo-EM refinement models correlated the best with experimental data. We then applied this sampling method to multi-domain F508del CFTR.

The F508del CFTR models presented here are compatible with RosettaLigand and we will leverage the rest of the Rosetta Software Suite for future rational CFTR drug design. Nevertheless, our models present several limitations to consider for drug design. Firstly, threading the F508del sequence onto the WT protein fails to account for V510 reorientation around the H3-H4 region (Supplemental Figure S4A), thus building this loop from the NBD1 crystal structure may offer this advantage. Our multi-domain models are still missing many loop regions that remain undetermined in the cryo-EM density. For example, the regulatory insertion (RI) region changes the thermodynamic stability of CFTR [56] and adopts distinct conformations, one of which has been postulated to lead to F508del unfolding [57]. Experimental evidence suggests removal of the RI significantly changes the folding trajectory of F508del, and thus excluding the RI region limits our interpretation of any observed unfolding. Our models are also missing the regulatory domain (a large unstructured 200 residues between NBD1 and TMD2), the glycosylation site, and the loop connecting TMD2 to NBD2. Modeling loop regions either with loop modeling in Rosetta or using the cryo-EM density and Rosetta enumerated sampling will further improve the biological relevance of our approach.

Our multi-domain F508del models presents advantages and limitations towards the goal of providing a basis for computer-based drug design. The inactive and active state F508del models successfully captured the thermodynamic instability of F508del CFTR evident by the overall higher Rosetta scores of these models compared to WT. However, R1070W destabilizes the protein structure in our models, but stabilized the NBD1/TMD2 interface suggesting that our models captured local energetic changes but failed to capture global changes. Corrector compounds stabilize both F508del NBD1 and the NBD1/TMD interface. Thus, modeling multi-domain F508del CFTR represents a key step towards structure-based drug design for CF. VX-809 stabilized F508del CFTR in the active state when included in our model. Thus, the active state and may offer more biologically relevant sampling with this technique than the inactive state. Given the sequestered R1070W and the occluded VX-809 binding pocket in the inactive conformation both increased the overall scores of our models, we speculate our method is limited at handling perturbations to the inside of the CFTR structure, and perhaps better suited to modeling surface level changes, such as small molecules bound to exposed binding pockets.

Beyond corrector compounds, our methods may be applied to design of potentiator compounds as well, which F508del CFTR requires for proper gating. Recent studies elucidated the binding site of the FDA approved potentiator Ivacaftor (VX-770) and experimental compound GLPG1837 to CFTR [14,15] and revealed overlapping binding sites for potentiator compounds in transmembrane helix 8 (TM8). The proposed mechanism for potentiation involves stabilization of a kink in TM8 [15]. This mechanism may be leveraged to focus on TM8 as a target for computational potentiator design using the template-based sampling method described here for both F508del and other rarer CFTR variants.

The F508del CFTR and VX-809 modeling approach developed here will aid future rational, structure-based drug development efforts for CF. Understanding the binding mechanism allows for a combination of computational and experimental optimization against the clinically relevant F508del CFTR. In addition to modeling VX-809 binding, we can also use previously publish HTS campaigns to study corrector binding against both WT and mutant CFTR [58]. Although most of these compounds did not go through FDA-approval, these data are useful to computationally screen using structural models that conserve predicted interactions within new chemical space. Using the predicted models of the WT and mutants, we can better understand the physical characteristics that account for efficacy differences, which we can apply in developing new corrector molecules. These structure-based studies include docking these potential correctors to WT and mutant models with pharmacophore mapping to investigate the protein-ligand interactions. From this, we can curate ultra-large compound libraries such as Enamine for compounds that maintain these interactions and achieve adequate potency against the mutant CFTR. Our group has developed approaches to perform computer-aided drug design in tandem with comparative modeling [53] and established methods for screening flexible ligands on ensembles of protein structure [54]. Thus, the RosettaCM approach used here will allow us to generate ensembles of mutant CFTR structures for virtual screening in a manner that preserves the known cryo-EM structural data and incorporates structural changes from mutations. This method will allow simulation of rare, currently untreatable CFTR variants and generate structural data for computational drug design towards the goal of personalized medicine for CF.

## Data Availability

A protocol capture on how to generate CFTR refinement models as well as CFTR comparative models is included in the Supplemental Information.

## Supplementary Materials

The following supporting information can be downloaded at: https://www.mdpi.com/article/10.3390/biom12030471/s1, Figure S1. Cryo-EM refinement parameter optimization, Figure S2. Cryo-EM refinement ensemble diversity, Figure S3. Testing RosettaCM template number against NBD1 CFTR thermodynamic data, Figure S4. Comparing RosettaCM F508del models to publish crystal NBD1 structures, Figure S5. RosettaCM F508del ensemble diversity. Figure S6. Domain-domain interface residues numbers, Figure S7. WT and F508del CFTR domain-domain interrace calculations, Figure S8. F508del/R1070W score versus RMSD plots, Figure S9. F508del/R1070W ensemble diversity, Figure S10. F508del/R1070W domain-domain interface calculations, Figure S11. F508del + VX-809 score versus RMSD plots, Figure S12. F508del + VX-809 ensemble diversity, Figure S13. F508del + VX-809 interface energy calculations, Figure S14. F508del/R1070W + VX-809 score versus RMSD plots, ensemble diversity, and interface energy calculations. Table S1. Model Fasta Sequences, Table S2. CFTR NBD1 Tm and ΔG thermodynamic data for testing against RosettaCM template number. Protocol capture also included in Supplemental Materials.

## References

[1] Cutting G.R. (2014). Cystic fibrosis genetics: From molecular understanding to clinical application. Nat. Rev. Genet..

[2] Liu F., Zhang Z., Csanády L., Gadsby D.C., Chen J. (2017). Molecular Structure of the Human CFTR Ion Channel. Cell.

[3] Riordan J.R., Rommens J.M., Kerem B., Alon N., Grzelczak Z., Zielenski J., Zielenski J., Lok S., Plavsic N., Chou J. (1989). Identification of the Cystic Fibrosis Gene: Cloning and Characterization of Complementary DNA. Science.

[4] Welsh M., Smith A.E. (1993). Molecular mechanisms of CFTR chloride channel dysfunction in cystic fibrosis. Cell.

[5] Koch C., Høiby N. (1993). Pathogenesis of cystic fibrosis. Lancet.

[6] Zhang Z., Liu F., Chen J. (2018). Molecular structure of the ATP-bound, phosphorylated human CFTR. Proc. Natl. Acad. Sci. USA.

[7] Van Goor F., Hadida S., Grootenhuis P.D.J., Burton B., Stack J.H., Straley K.S., Decker C.J., Miller M., McCartney J., Olson E.R. (2011). Correction of the F508del-CFTR protein processing defect in vitro by the investigational drug VX-809. Proc. Natl. Acad. Sci. USA.

[8] Van Goor F. (2009). Rescue of CF airway epithelial cell functino in vitro by a CFTR potentiator, VX-770. Proc. Natl. Acad. Sci. USA.

[9] Pinto M.C., AL Silva I., Figueira M.F., Amaral M.D., Lopes-Pacheco M. (2021). Pharmacological Modulation of Ion Channels for the Treatment of Cystic Fibrosis. J. Exp. Pharmacol..

[10] Guerra L., Favia M., Di Gioia S., Laselva O., Bisogno A., Casavola V., Colombo C., Conese M. (2020). The preclinical discovery and development of the combination of ivacaftor + tezacaftor used to treat cystic fibrosis. Expert Opin. Drug Discov..

[11] Jain R., Kazmerski T.M., Aitken M.L., West N., Wilson A., Bozkanat K.M., Montemayor K., von Berg K., Sjoberg J., Poranski M. (2021). Challenges Faced by Women with Cystic Fibrosis. Clin. Chest Med..

[12] Rotolo S.M., Duehlmeyer S., Slack S.M., Jacobs H.R., Heckman B. (2020). Testicular pain following initiation of elexacaftor/tezacaftor/ivacaftor in males with cystic fibrosis. J. Cyst. Fibros..

[13] Dagenais R.V.E., Su V.C.H., Quon B.S. (2020). Real-World Safety of CFTR Modulators in the Treatment of Cystic Fibrosis: A Systematic Review. J. Clin. Med..

[14] Laselva O., Qureshi Z., Zeng Z.-W., Petrotchenko E.V., Ramjeesingh M., Hamilton C.M., Huan L.-J., Borchers C.H., Pomès R., Young R. (2021). Identification of binding sites for ivacaftor on the cystic fibrosis transmembrane conductance regulator. iScience.

[15] Liu F., Zhang Z., Levit A., Levring J., Touhara K.K., Shoichet B.K., Chen J. (2019). Structural identification of a hotspot on CFTR for potentiation. Science.

[16] Fiedorczuk K., Chen J. (2022). Mechanism of CFTR correction by type I folding correctors. Cell.

[17] Baatallah N., Elbahnsi A., Mornon J.-P., Chevalier B., Pranke I., Servel N., Zelli R., Décout J.-L., Edelman A., Sermet-Gaudelus I. (2021). Pharmacological chaperones improve intra-domain stability and inter-domain assembly via distinct binding sites to rescue misfolded CFTR. Cell. Mol. Life Sci..

[18] Okiyoneda T., Veit G., Dekkers J.F., Bagdany M., Soya N., Xu H., Roldan A., Verkman A.S., Kurth M.J., Simon A. (2013). Mechanism-based corrector combination restores ΔF508-CFTR folding and function. Nat. Chem. Biol..

[19] Veit G., Roldan A., Hancock M.A., Da Fonte D.F., Xu H., Hussein M., Frenkiel S., Matouk E., Velkov T., Lukacs G.L. (2020). Allosteric folding correction of F508del and rare CFTR mutants by elexacaftor-tezacaftor-ivacaftor (Trikafta) combination. JCI Insight.

[20] Rabeh W., Bossard F., Xu H., Okiyoneda T., Bagdany M., Mulvihill C.M., Du K., di Bernardo S., Liu Y., Konermann L. (2012). Correction of Both NBD1 Energetics and Domain Interface Is Required to Restore ΔF508 CFTR Folding and Function. Cell.

[21] Mendoza J.L., Schmidt A., Li Q., Nuvaga E., Barrett T., Bridges R.J., Feranchak A.P., Brautigam C.A., Thomas P.J. (2012). Requirements for Efficient Correction of ΔF508 CFTR Revealed by Analyses of Evolved Sequences. Cell.

[22] Laselva O., Molinski S., Casavola V., Bear C.E. (2018). Correctors of the Major Cystic Fibrosis Mutant Interact through Membrane-Spanning Domains. Mol. Pharmacol..

[23] Hudson R.P., Dawson J.E., Chong P.A., Yang Z., Millen L., Thomas P.J., Brouillette C.G., Forman-Kay J.D. (2017). Direct binding of the Corrector VX-809 to Human CFTR NBD1: Evidence of an Allosteric coupling between the Binding site and the NBD1:CL4 Interface. Mol. Pharmacol..

[24] Bahia M.S., Khazanov N., Zhou Q., Yang Z., Wang C., Hong J.S., Rab A., Sorscher E.J., Brouillette C.G., Hunt J.F. (2021). Stability Prediction for Mutations in the Cytosolic Domains of Cystic Fibrosis Transmembrane Conductance Regulator. J. Chem. Inf. Model..

[25] Estácio S.G., Martiniano H.F.M.C., Faísca P.F.N. (2016). Thermal unfolding simulations of NBD1 domain variants reveal structural motifs associated with the impaired folding of F508del-CFTR. Mol. BioSyst..

[26] Zhenin M., Noy E., Senderowitz H. (2015). REMD Simulations Reveal the Dynamic Profile and Mechanism of Action of Deleterious, Rescuing, and Stabilizing Perturbations to NBD1 from CFTR. J. Chem. Inf. Model..

[27] Abreu B., Lopes E.F., Oliveira A.S.F., Soares C.M. (2019). F508del disturbs the dynamics of the nucleotide binding domains of CFTR before and after ATP hydrolysis. Proteins Struct. Funct. Bioinform..

[28] Odera M., Furuta T., Sohma Y., Sakurai M. (2018). Molecular dynamics simulation study on the structural instability of the most common cystic fibrosis-associated mutant ΔF508-CFTR. Biophys. Phys..

[29] Wang R.Y.R., Song Y., Barad B.A., Cheng Y., Fraser J.S., DiMaio F. (2016). Automated structure refinement of macromolecular assemblies from cryo-EM maps using Rosetta. eLife.

[30] Song Y., DiMaio F., Wang R.Y.-R., Kim D., Miles C., Brunette T., Thompson J., Baker D. (2013). High-Resolution Comparative Modeling with RosettaCM. Structure.

[31] Protasevich I., Yang Z., Wang C., Atwell S., Zhao X., Emtage S., Wetmore D., Hunt J.F., Brouillette C.G. (2010). Thermal unfolding studies show the disease causing F508del mutation in CFTR thermodynamically destabilizes nucleotide-binding domain 1. Protein Sci..

[32] Farinha C.M., Canato S. (2017). From the endoplasmic reticulum to the plasma membrane: Mechanisms of CFTR folding and trafficking. Cell. Mol. Life Sci..

[33] Du K., Sharma M., Lukacs G. (2004). The ΔF508 cystic fibrosis mutation impairs domain-domain interactions and arrests post-translational folding of CFTR. Nat. Struct. Mol. Biol..

[34] Hwang T.-C., Yeh J.-T., Zhang J., Yu Y.-C., Yeh H.-I., Destefano S. (2018). Structural mechanisms of CFTR function and dysfunction. J. Gen. Physiol..

[35] Yarov-Yarovoy V., Schonbrun J., Baker D. (2005). Multipass membrane protein structure prediction using Rosetta. Proteins Struct. Funct. Bioinform..

[36] Barth P., Schonbrun J., Baker D. (2007). Toward high-resolution prediction and design of transmembrane helical protein structures. Proc. Natl. Acad. Sci. USA.

[37] Viklund H., Elofsson A. (2008). OCTOPUS: Improving topology prediction by two-track ANN-based preference scores and an extended topological grammar. Bioinformatics.

[38] Trott O., Olson A.J. (2010). AutoDock Vina: Improving the speed and accuracy of docking with a new scoring function, efficient optimization, and multithreading. J. Comput. Chem..

[39] Mendenhall J., Brown B.P., Kothiwale S., Meiler J. (2020). BCL::Conf: Improved Open-Source Knowledge-Based Conformation Sampling Using the Crystallography Open Database. J. Chem. Inf. Model..

[40] Lemmon G., Meiler J., Baron R. (2012). Rosetta Ligand Docking with Flexible XML Protocols BT–Computational Drug Discovery and Design.

[41] Bender B.J., Marlow B., Meiler J. (2020). Improving homology modeling from low-sequence identity templates in Rosetta: A case study in GPCRs. PLoS Comput. Biol..

[42] Farkas B., Tordai H., Padányi R., Tordai A., Gera J., Paragi G., Hegedűs T. (2019). Discovering the chloride pathway in the CFTR channel. Cell. Mol. Life Sci..

[43] Aleksandrov A.A., Kota P., Cui L., Jensen T., Alekseev A.E., Reyes S., He L., Gentzsch M., Aleksandrov L.A., Dokholyan N.V. (2012). Allosteric Modulation Balances Thermodynamic Stability and Restores Function of ΔF508 CFTR. J. Mol. Biol..

[44] Denning G., Anderson M., Amara J.F., Marshall J., Smith A.E., Welsh M. (1992). Processing of mutant cystic fibrosis transmembrane conductance regulator is temperature-sensitive. Nature.

[45] Lewis H.A., Zhao X., Wang C., Sauder J.M., Rooney I., Noland B.W., Lorimer D., Kearins M.C., Conners K., Condon B. (2005). Impact of the ΔF508 Mutation in First Nucleotide-binding Domain of Human Cystic Fibrosis Transmembrane Conductance Regulator on Domain Folding and Structure. J. Biol. Chem..

[46] Lewis H., Wang C., Zhao X., Hamuro Y., Conners K., Kearins M., Lu F., Sauder J., Molnar K., Coales S. (2010). Structure and Dynamics of NBD1 from CFTR Characterized Using Crystallography and Hydrogen/Deuterium Exchange Mass Spectrometry. J. Mol. Biol..

[47] Khushoo A., Yang Z., Johnson A.E., Skach W.R. (2011). Ligand-Driven Vectorial Folding of Ribosome-Bound Human CFTR NBD1. Mol. Cell.

[48] Thibodeau P., Richardson J.M., Wang W., Millen L., Watson J., Mendoza J., Du K., Fischman S., Senderowitz H., Lukacs G. (2010). The Cystic Fibrosis-causing Mutation ΔF508 Affects Multiple Steps in Cystic Fibrosis Transmembrane Conductance Regulator Biogenesis. J. Biol. Chem..

[49] He L., Kota P., Aleksandrov A.A., Cui L., Jensen T., Dokholyan N.V., Riordan J.R. (2013). Correctors of ΔF508 CFTR restore global conformational maturation without thermally stabilizing the mutant protein. FASEB J..

[50] Lopes-Pacheco M., Silva I.A., Turner M.J., Carlile G.W., Sondo E., Thomas D.Y., Pedemonte N., Hanrahan J.W., Amaral M.D. (2020). Characterization of the mechanism of action of RDR01752, a novel corrector of F508del-CFTR. Biochem. Pharmacol..

[51] Loo T.W., Bartlett M.C., Clarke D.M. (2013). Corrector VX-809 stabilizes the first transmembrane domain of CFTR. Biochem. Pharmacol..

[52] Eckford P.D., Ramjeesingh M., Molinski S., Pasyk S., Dekkers J.F., Li C., Ahmadi S., Ip W., Chung T.E., Du K. (2014). VX-809 and Related Corrector Compounds Exhibit Secondary Activity Stabilizing Active F508del-CFTR after Its Partial Rescue to the Cell Surface. Chem. Biol..

[53] Combs S.A., DeLuca S.L., DeLuca S.H., Lemmon G., Nannemann D., Nguyen E., Willis J.R., Sheehan J.H., Meiler J. (2013). Small-molecule ligand docking into comparative models with Rosetta. Nat. Protoc..

[54] DeLuca S., Khar K., Meiler J. (2015). Fully Flexible Docking of Medium Sized Ligand Libraries with RosettaLigand. PLoS ONE.

[55] Brown B.P., Mendenhall J., Geanes A.R., Meiler J. (2021). General Purpose Structure-Based Drug Discovery Neural Network Score Functions with Human-Interpretable Pharmacophore Maps. J. Chem. Inf. Model..

[56] Aleksandrov A.A., Kota P., Aleksandrov L.A., He L., Jensen T., Cui L., Gentzsch M., Dokholyan N.V., Riordan J.R. (2010). Regulatory Insertion Removal Restores Maturation, Stability and Function of ΔF508 CFTR. J. Mol. Biol..

[57] Scholl D., Sigoillot M., Overtus M., Martinez R.C., Martens C., Wang Y., Pardon E., Laeremans T., Garcia-Pino A., Steyaert J. (2021). A topological switch in CFTR modulates channel activity and sensitivity to unfolding. Nat. Chem. Biol..

[58] Veit G., Xu H., Dreano E., Avramescu R.G., Bagdany M., Beitel L.K., Roldan A., Hancock M., Lay C., Li W. (2018). Structure-guided combination therapy to potently improve the function of mutant CFTRs. Nat. Med..

