# Supplementary material for: Structural Comparative Modeling of Multi-Domain F508del CFTR
Source: Biomolecules. 2022 Mar 18;12(3):471. doi: 10.3390/biom12030471 (PMC8946492; doi:10.3390/biom12030471)
Supplement: Supplementary file 1 [file biomolecules-12-00471-s001.zip › biomolecules-1621250-supplementary.pdf]

SUPPORTING INFORMATION

for

Structural comparative modeling of multi-domain F508del CFTR

Eli Fritz McDonald[1,2], Hope Woods[2,3], Shannon Smith[2,3], Minsoo Kim[3], Clara T. Schoeder[1,2,4], Lars Plate[1,5], Jens Meiler[1,2,4,6,7,*]

[1]Department of Chemistry, Vanderbilt University, Nashville, TN, United States of America

[2]Center for Structural Biology, Vanderbilt University, Nashville, TN, United States of America

[3]Program in Chemical and Physical Biology, Vanderbilt University, Nashville, TN, United States of America

[4]University Leipzig Medical School, Leipzig University, Leipzig, Saxony, Germany

[5]Department of Biological Sciences, Vanderbilt University, Nashville, TN, United States of America

[6]Department of Pharmacology, Vanderbilt University, Nashville, TN, United States of America

[7]Institute for Drug Discovery, Leipzig University, Leipzig, Saxony, Germany

TABLE OF CONTENT

Supplemental Figures S1 – S14 pp 2-17

Supplemental Tables S1-S2 pp 18-20

Supporting Information References p 21

Protocol Capture p 22-31

SUPPLEMENTAL FIGURE S1

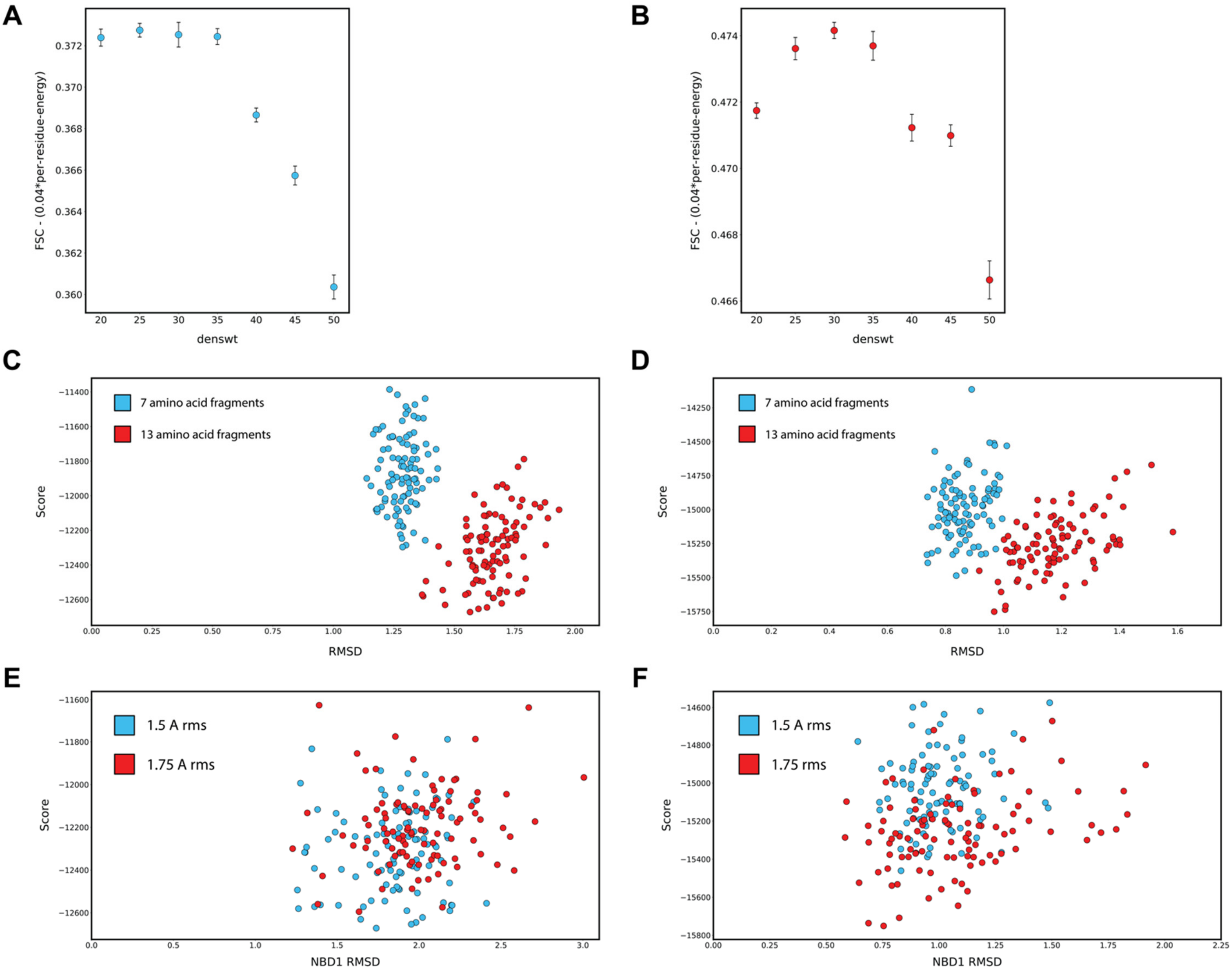

Supplemental Figure S1. Cryo-EM refinement parameter optimization. A. Increasing weight put on cryo-EM density by refinement algorithm (denswt) vs. Fourier Shell Coefficient (FSC) minus 0.04 times the per-residue energy score for 100 5UAK (1) refined models generated at each denswt. Error bars represent standard error of the mean. Score is shown in REU. **B.** Increasing weight put on cryo-EM density by refinement algorithm (denswt) vs. Fourier Shell Coefficient (FSC) minus 0.04 times the per-residue energy score for 100 6MSM (2) refined models generated at each denswt. Error bars represent standard error of the mean. **C.** Cα RMSD vs. score plot of 100 5UAK refined models at a denswt of 35 using 7 (blue) or 13 (red) residue long fragments for insertion. **D.** Cα RMSD vs. score plot of 100 6MSM refined models at a denswt of 35 using 7 (blue) or 13 (red) residue long fragments for insertion. **E.** Cα NBD1 RMSD vs. score plot of 100 5UAK refined models at a denswt of 35,

13 residue long fragments, altering root mean squared (rms) distance of fragment insertion from 1.5 Å to 1.75 Å. Larger rms values (not shown for clarity) offer now improvement over 1.75 Å. **F.** Cα NBD1 RMSD vs. score plot of 100 6MSM refined models at a denswt of 35, 13 residue long fragments, altering root mean squared (rms) distance of fragment insertion from 1.5 Å to 1.75 Å. Larger rms values (not shown for clarity) offer now improvement over 1.75 Å.

SUPPLEMENTAL FIGURE S2

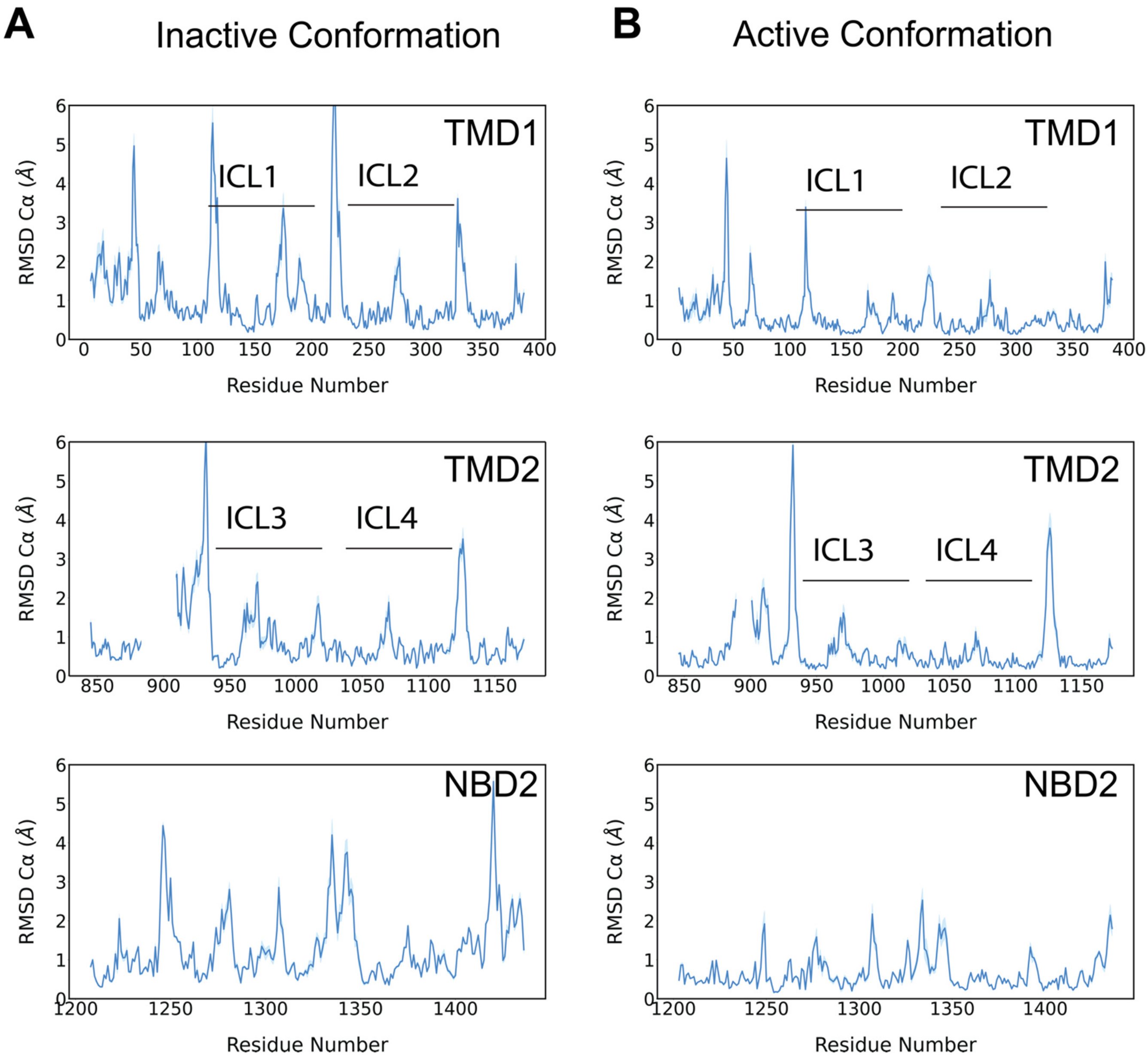

Supplemental Figure S2. Cryo-EM refinement ensemble diversity. A. Average residue Cα RMSD relative to the published model of the lowest scoring 100 inactive state WT refinement models for TMD1, TMD2, and NBD2. **B**. Average residue Cα RMSD relative to the published model of the lowest scoring 100 active state WT refinement models for TMD1, TMD2, and NBD2.

SUPPLEMENTAL FIGURE S3

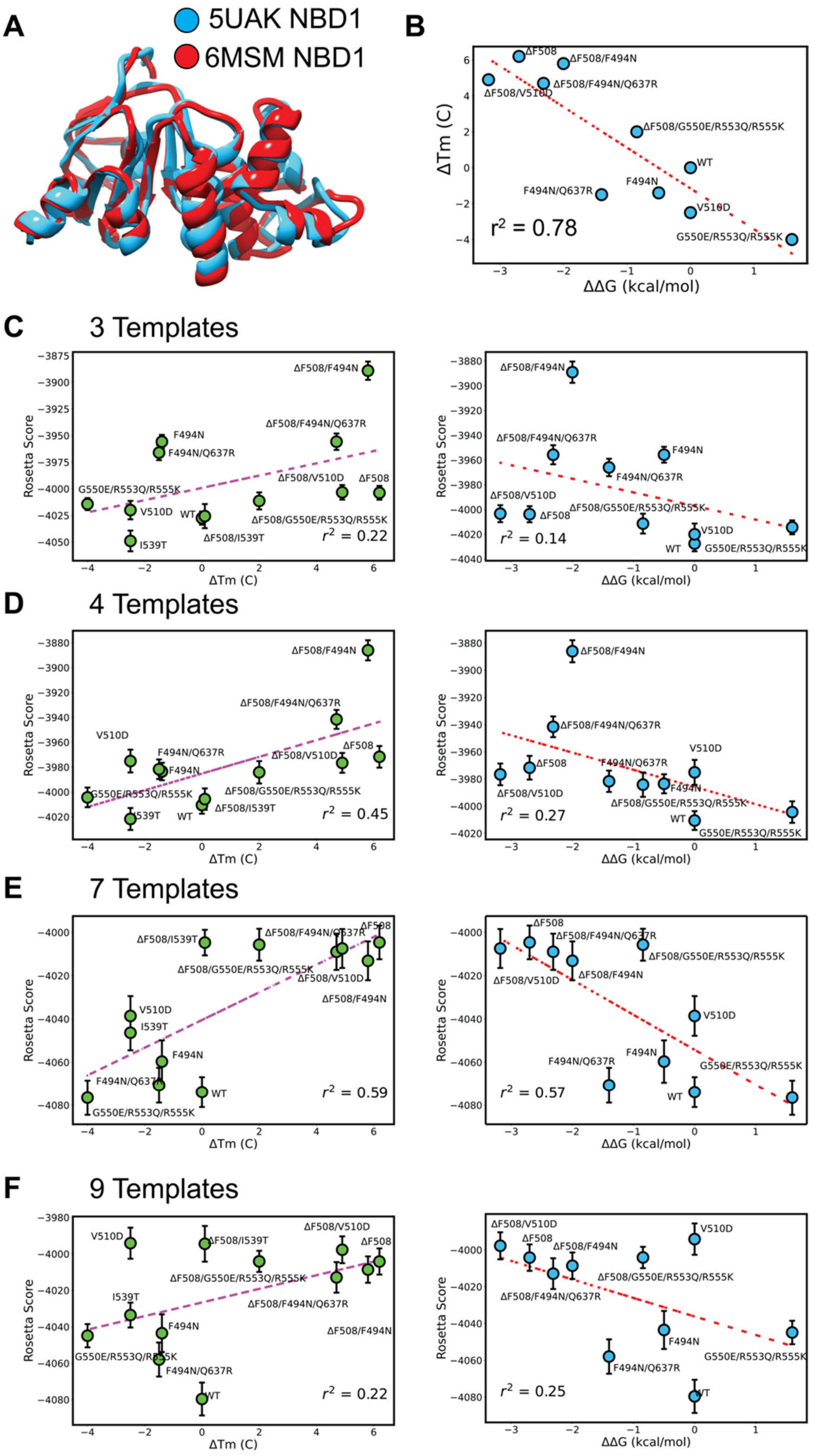

Supplemental Figure S3. Testing RosettaCM template number against NBD1 CFTR thermodynamic data.

A. 5UAK and 6MSM NBD1 (residues 385-402 and 439-637) aligned, RMSD between two domains is 2.231 Å.

B. Correlation between experimental $\Delta\Delta G$ and ΔT_M data from literature (3–5) (**Supplemental Table S2**). R squared values represent Pearson correlation coefficient and error bars represent standard error of the mean. Of note, error in experimental data likely ranges with +/- 1-2 kcal/mol. **C.** Correlation between Rosetta score in REU and ΔT_M (right) and $\Delta\Delta G$ (left) using three refinement templates for RosettaCM. **D.** Correlation between Rosetta score and ΔT_M (right) and $\Delta\Delta G$ (left) using four refinement templates for RosettaCM. **E.** Correlation between Rosetta score and ΔT_M (right) and $\Delta\Delta G$ (left) using seven refinement templates for RosettaCM. **F.** Correlation between Rosetta score and ΔT_M (right) and $\Delta\Delta G$ (left) using nine refinement templates for RosettaCM.

SUPPLEMENTAL FIGURE S4

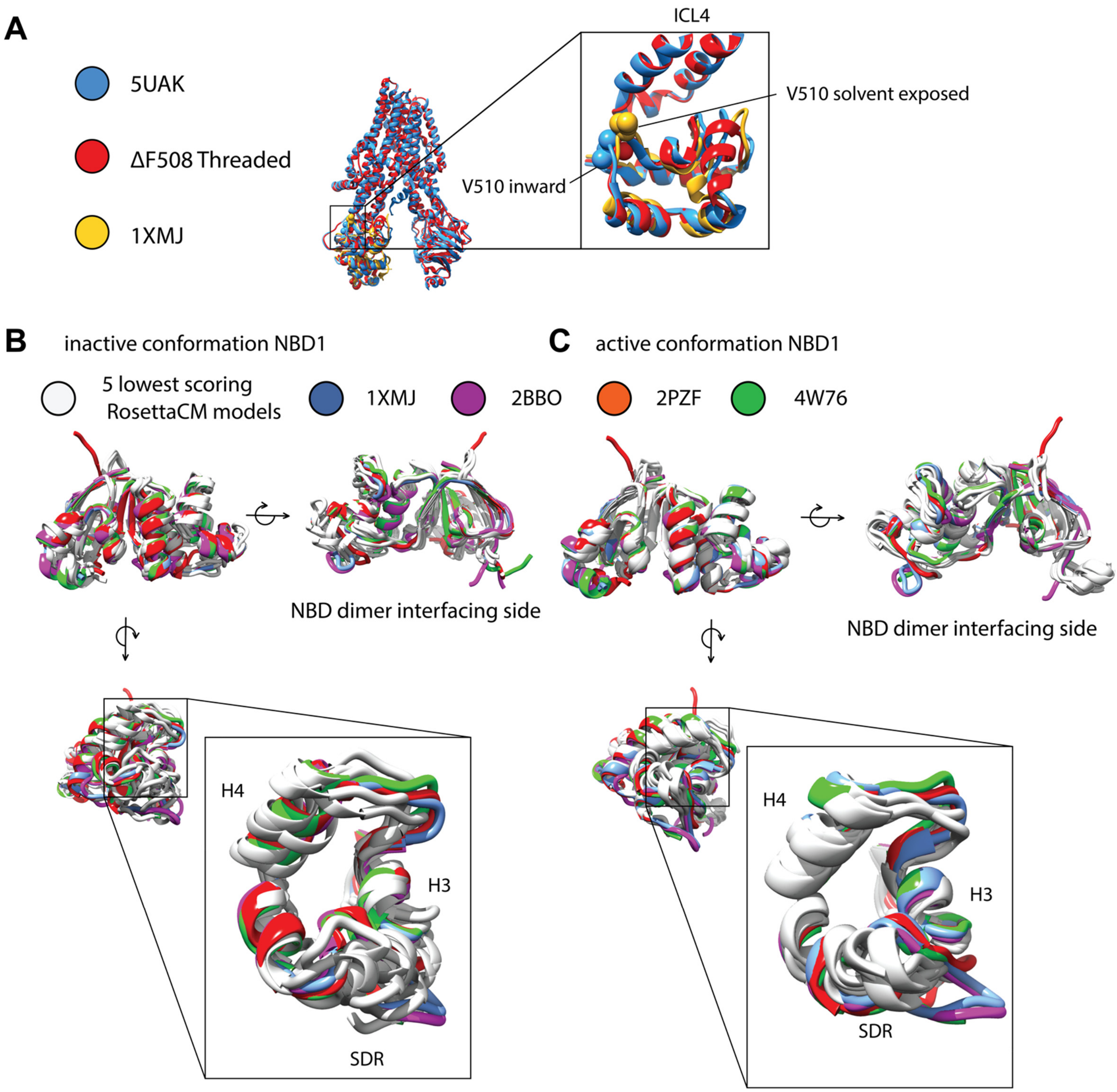

Supplemental Figure S4. Comparing RosettaCM F508del models to publish crystal NBD1 structures. A. In the 1XMJ crystal structure, residue V510 in the alpha helical subdomain shift from an "inward facing" to a solvent exposed orientation (gold). By contrast, threading the F508del sequence onto WT refined models fails to cause this shift (compare red and blue to gold). **B.** NBD1 of the lowest scoring five inactive F508del CFTR Rosetta CM

models superimposed onto F508del NBD1 crystal structures 1XMJ, 2BBO, 2PZF, 4W76. Residues 385-402 and 439-645 are depicted. The Helix 3 (H3) – Helix 4 (H4) loop and structurally diverse region (SDR) are highlighted because the RosettaCM models deviate from the crystal structures, likely due to the inclusion of multi-domain modeling versus single domain crystallization. The SDR in the inactive conformation demonstrates how well this region is sampled due to the low density of this region in the cryo-EM map. **C.** NBD1 of the lowest scoring five active F508del CFTR Rosetta CM models again superimposed onto F508del NBD1 crystal structures 1XMJ, 2BBO, 2PZF, 4W76. Residues 385-409 and 435-637 are depicted. The Helix 3 (H3) – Helix 4 (H4) loop and structurally diverse region (SDR) are again highlighted in the inset to demonstrate perturbations in these regions in the multi-domain models compared to the single domain crystal structure.

SUPPLEMENTAL FIGURE S5

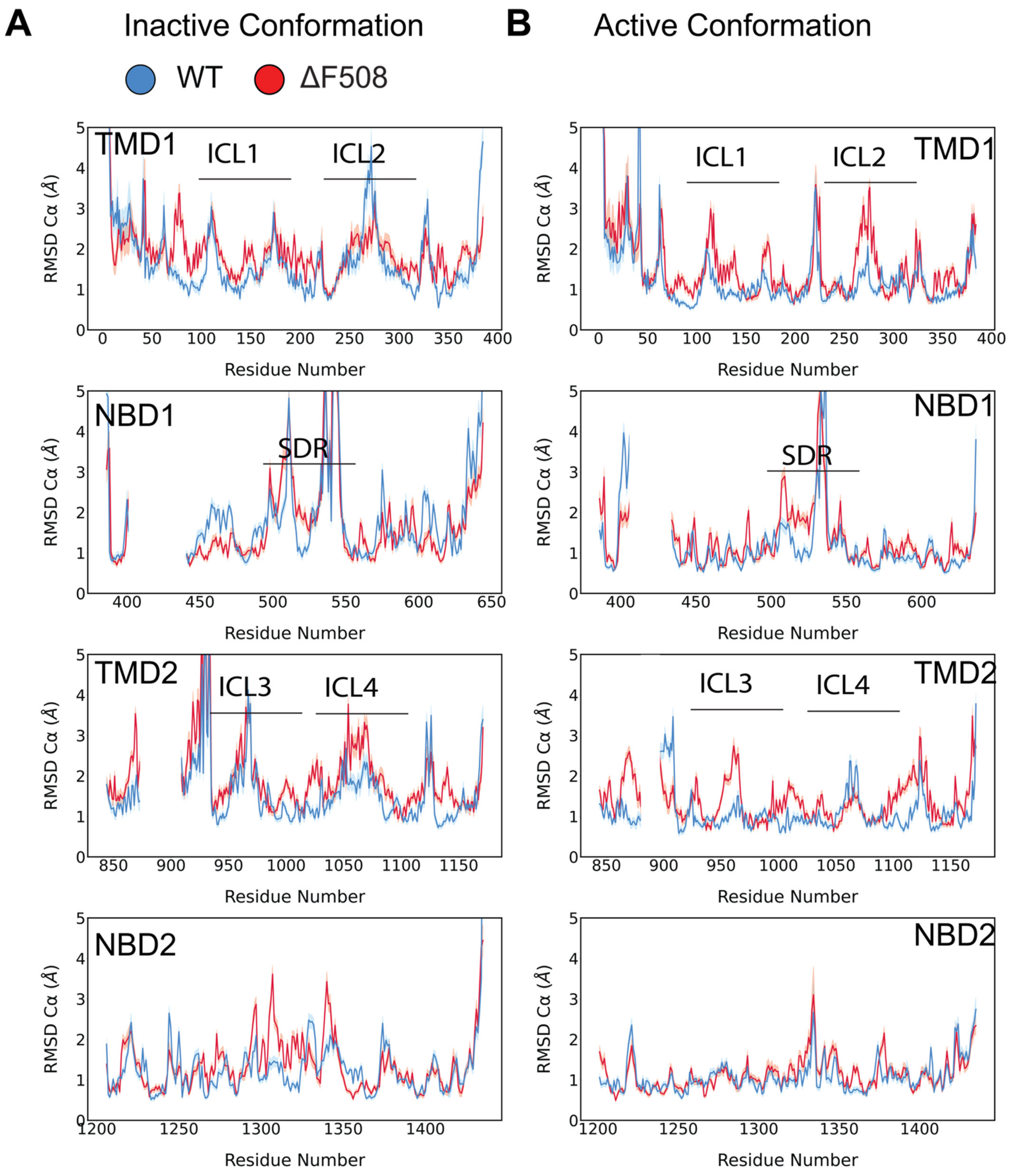

Supplemental Figure S5. . RosettaCM F508del ensemble diversity. A. Average residue Cα RMSD of the lowest scoring 100 inactive state WT (blue) and F508del (red) models vs. residue number for TMD1, NBD1,

TMD2, and NBD2. **B**. Average residue Cα RMSD of the lowest scoring 100 active state WT (blue) and F508del (red) models vs. residue number for TMD1, NBD1, TMD2, and NBD2.

SUPPLEMENTAL FIGURE S6

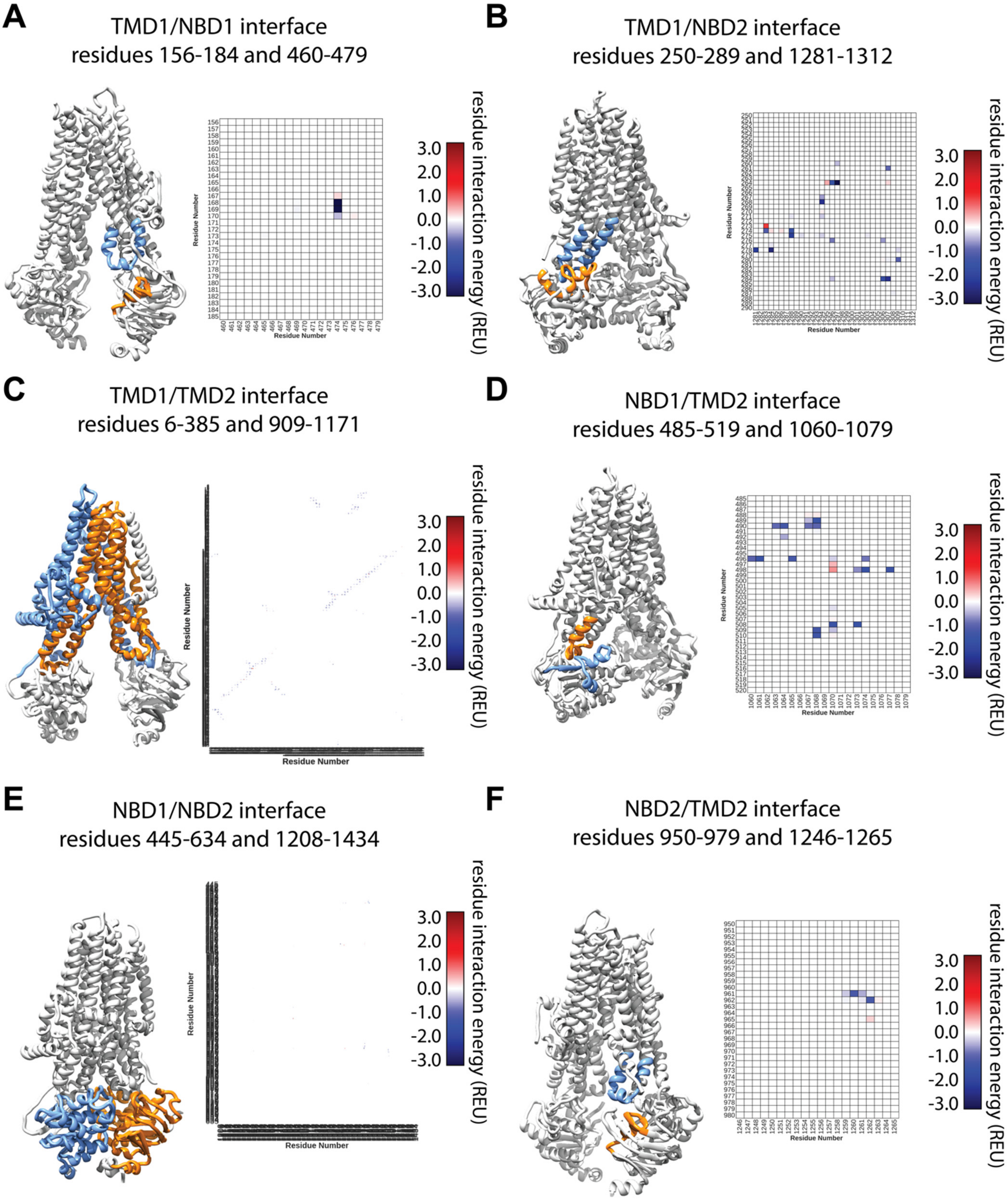

Supplemental Figure S6. Domain-domain interface residues numbers. A. The TMD1/NBD1 interface is calculated by considering the interactions between residues 156-184 in TMD1 (blue) and residues 460-479 in

NBD1 (orange). An example heatmap between the corresponding residues is shown. **B**. The TMD1/NBD2 interface is calculated by considering the interactions between residues 250-289 in TMD1 (blue) and residues 1281-1312 in NBD2 (orange). **C.** The TMD1/TMD2 interface is calculated by considering the interactions between most residues in both domains as the interface is extensive. We considered residues 6-385 in TMD1 (blue) and residues 909-1171 in TMD2 (orange). **D.** The NBD1/TMD2 interface is calculated by considering the interactions between residues 485-519 in NBD1 (blue) and residues 1060-1079 in TMD2 (orange). **E.** The NBD1/NBD2 interface is calculated by considering the interactions between most residues in both domains as the interface is extensive in the active conformation (shown), however the domains are not dimerized in the inactive conformation. We considered residues 445-635 in NBD1 (blue) and residues 1298-1435 in NBD2 (orange). **F.** The TMD2/NBD2 interface is calculated by considering the interactions between residues 950-980 in TMD2 (blue) and residues 1246-1266 in NBD2 (orange).

SUPPLEMENTAL FIGURE S7

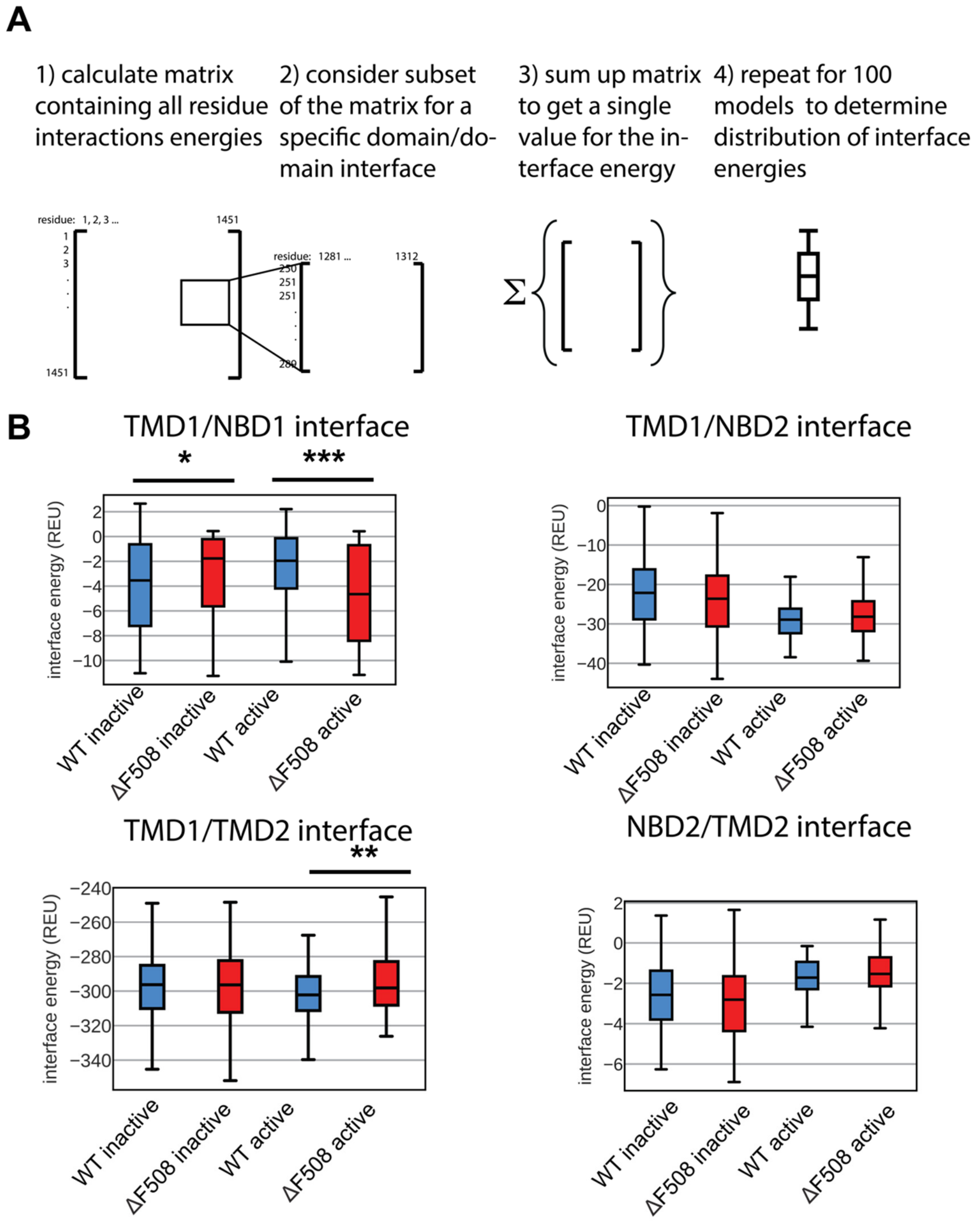

Supplemental Figure S7. WT and F508del CFTR domain-domain interrace calculations. A. Method for quantifying the distribution of the interface energy for each domain/domain interface. **B.** Quantification of the residue-residue interactions at each domain-domain interface across the lowest scoring 100 models between WT and F508del. (* - $p< 0.05$, ** - $p < 0.01$, *** - $p < 0.001$, **** - $p < 0.0001$). Statistical significance was calculated with a Mann-Whitney U test.

SUPPLEMENTAL FIGURE S8

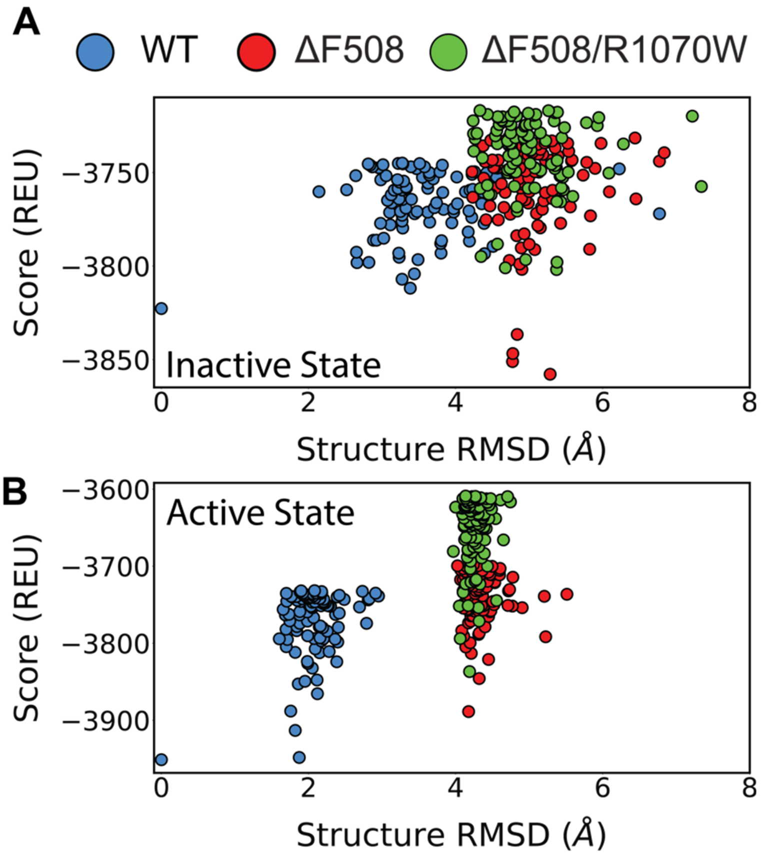

Supplemental Figure S8. F508del/R1070W score versus RMSD plots.A. Cα RMSD vs. score plot of the lowest scoring 100 inactive conformation models from ensembles of WT (blue) and F508del (red), and F508del/R1070W (green) CFTR. RMSD is calculated relative to the lowest scoring WT model. Score is shown in REU. **B.** Cα RMSD vs. score plot of the lowest scoring 100 active conformation models from ensembles of WT (blue) and F508del (red), and F508del/R1070W (green) CFTR. RMSD is calculated relative to the lowest scoring WT model.

SUPPLEMENTAL FIGURE S9

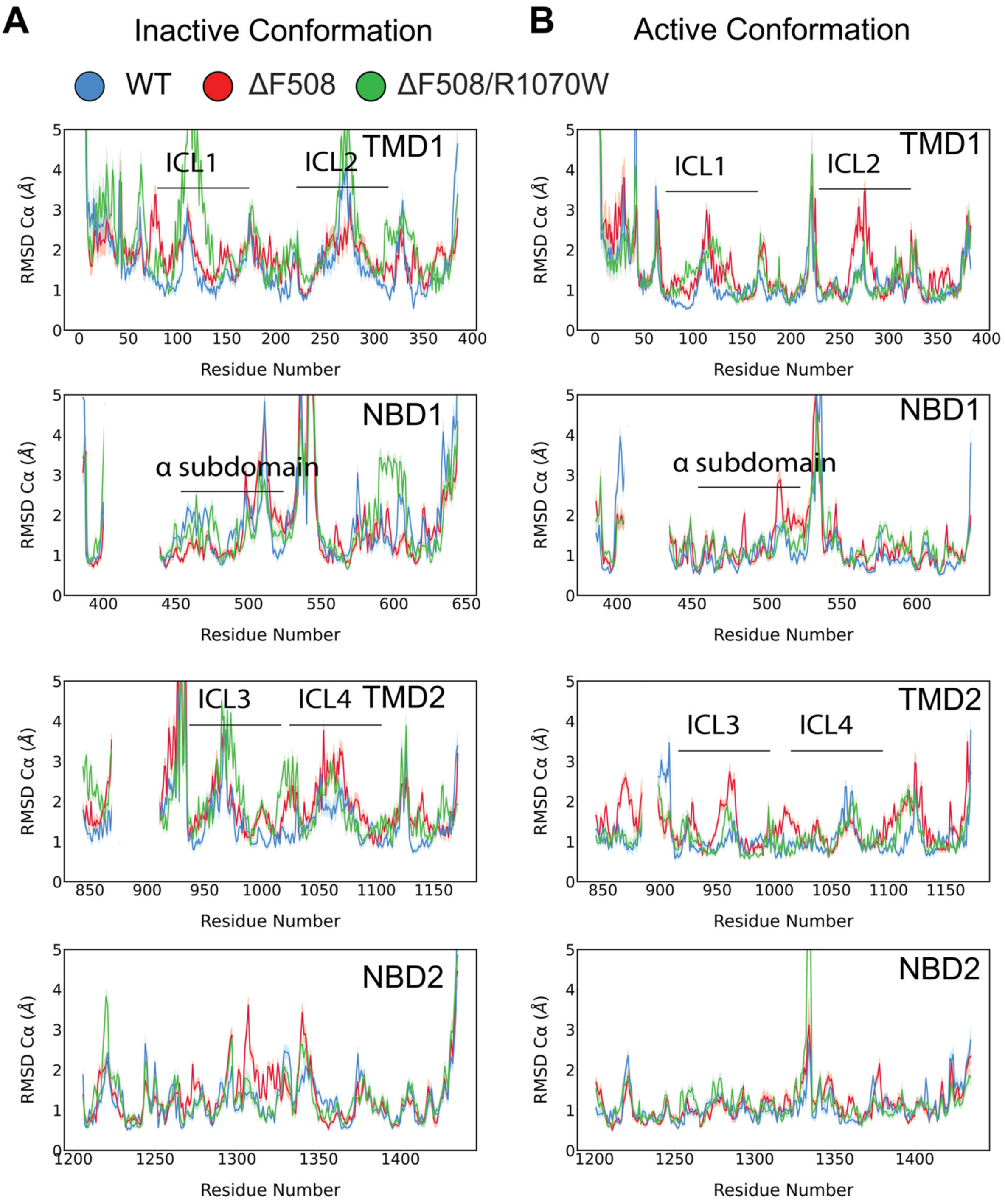

Supplemental Figure S9. F508del/R1070W ensemble diversity. A. Average residue Cα RMSD of the lowest scoring 100 inactive state WT (blue), F508del (red), and F508del/R1070W (green) models vs. residue number for TMD1, NBD1, TMD2, and NBD2. **B**. Average residue Cα RMSD of the lowest scoring 100 active state WT

(blue), F508del (red), and F508del/R1070W (green) models vs. residue number for TMD1, NBD1, TMD2, and NBD2.

SUPPLEMENTAL FIGURE S10

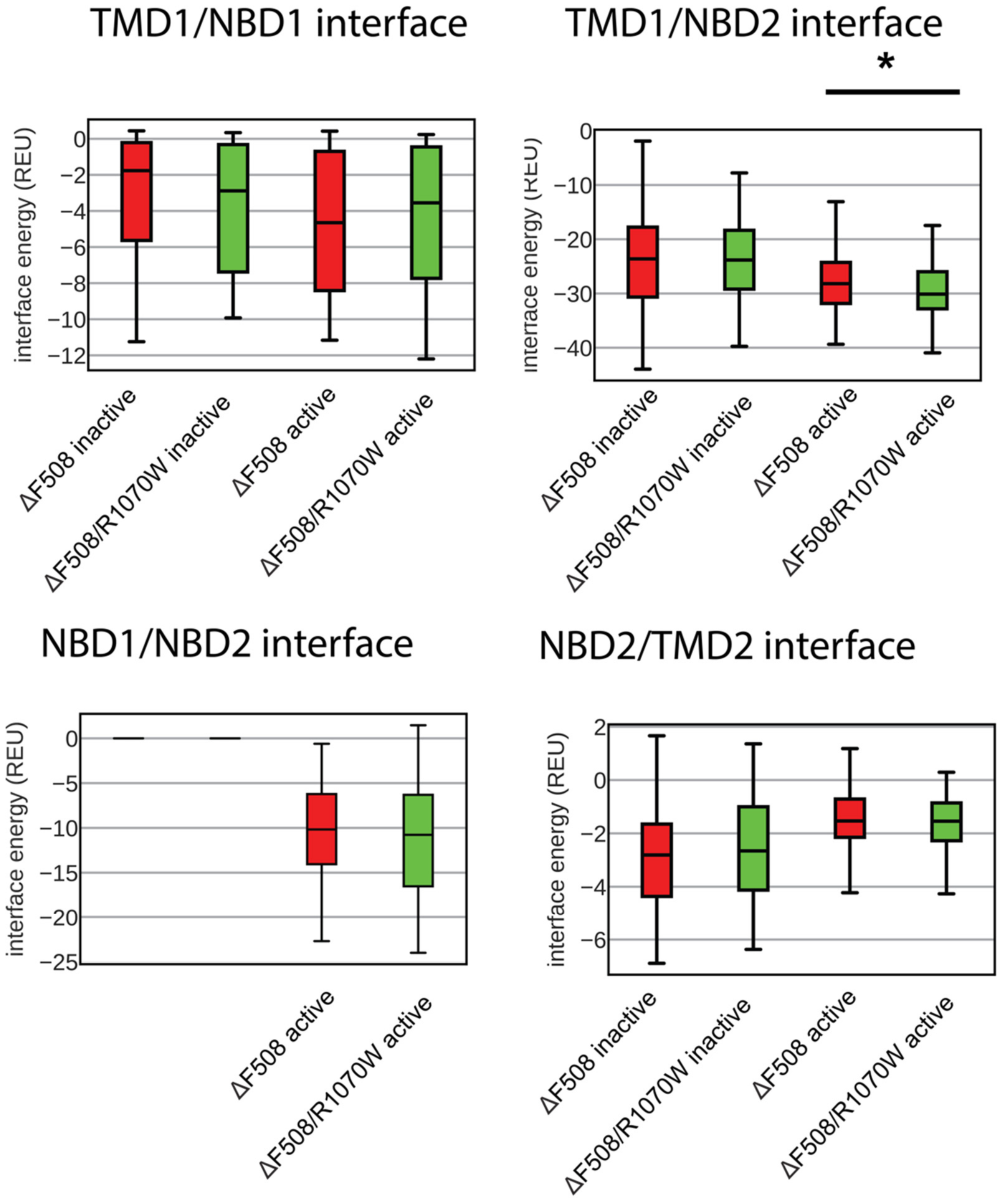

Supplemental Figure S10. F508del/R1070W domain-domain interface calculations. Quantification of the residue-residue interactions at each domain-domain interface across the lowest scoring 100 models between F508del and F508del/R1070W. (* - $p < 0.05$, ** - $p < 0.01$, *** - $p < 0.001$, **** - $p < 0.0001$). Statistical significance was calculated with a Mann-Whitney U test.

SUPPLEMENTAL FIGURE S11

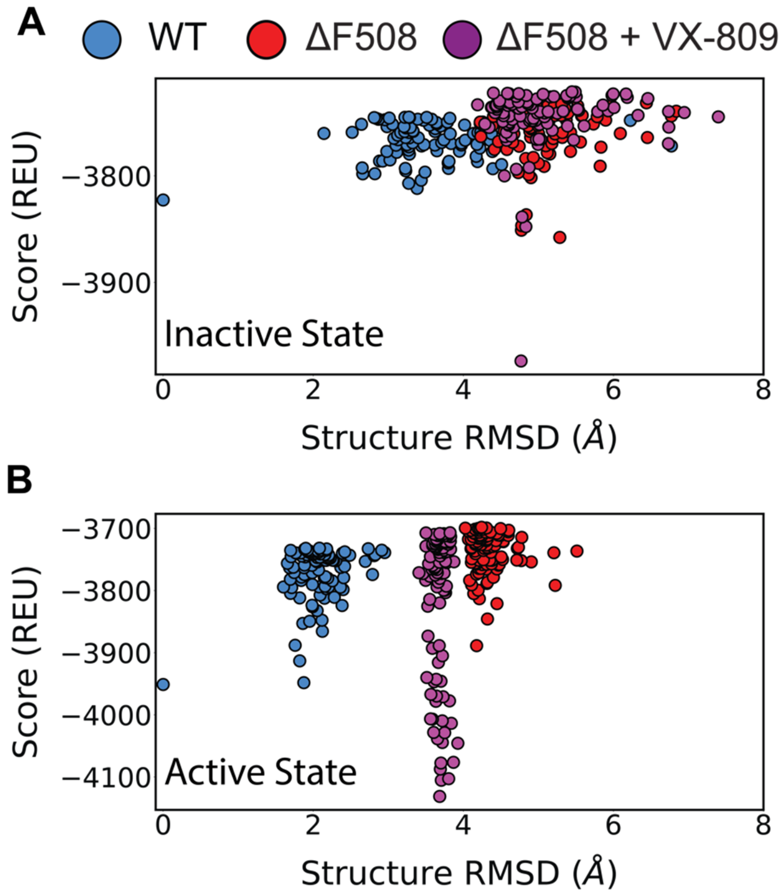

Supplemental Figure S11. F508del + VX-809 score versus RMSD plots. A. Cα RMSD vs. score plot of the lowest scoring 100 inactive conformation models from ensembles of WT (blue) and F508del (red), and F508del + VX-809 (purple). RMSD is calculated relative to the lowest scoring WT model. Score is shown in REU. **B.** Cα RMSD vs. score plot of the lowest scoring 100 active conformation models from ensembles of WT (blue) and F508del (red), and F508del + VX-809 (purple). RMSD is calculated relative to the lowest scoring WT model.

SUPPLEMENTAL FIGURE S12

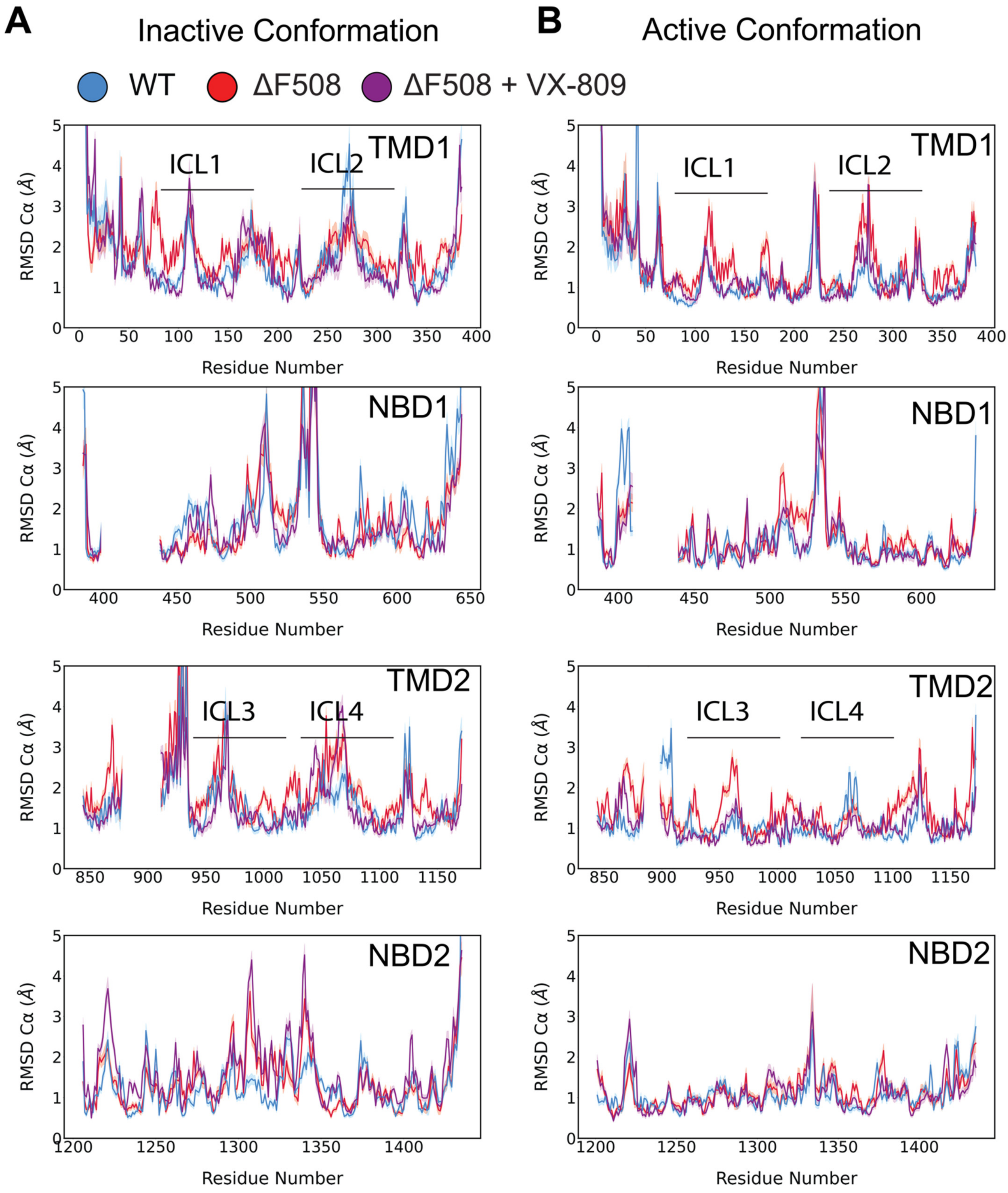

Supplemental Figure S12. F508del + VX-809 ensemble diversity. Average residue Cα RMSD of the lowest scoring 100 inactive state WT (blue), F508del (red), and F508del +VX-809 (purple) models vs. residue number

for TMD1, NBD1, TMD2, and NBD2. **B**. Average residue Cα RMSD of the lowest scoring 100 active state WT (blue), F508del (red), and F508del +VX-809 (purple) models vs. residue number for TMD1, NBD1, TMD2, and NBD2.

SUPPLEMENTAL FIGURE S13

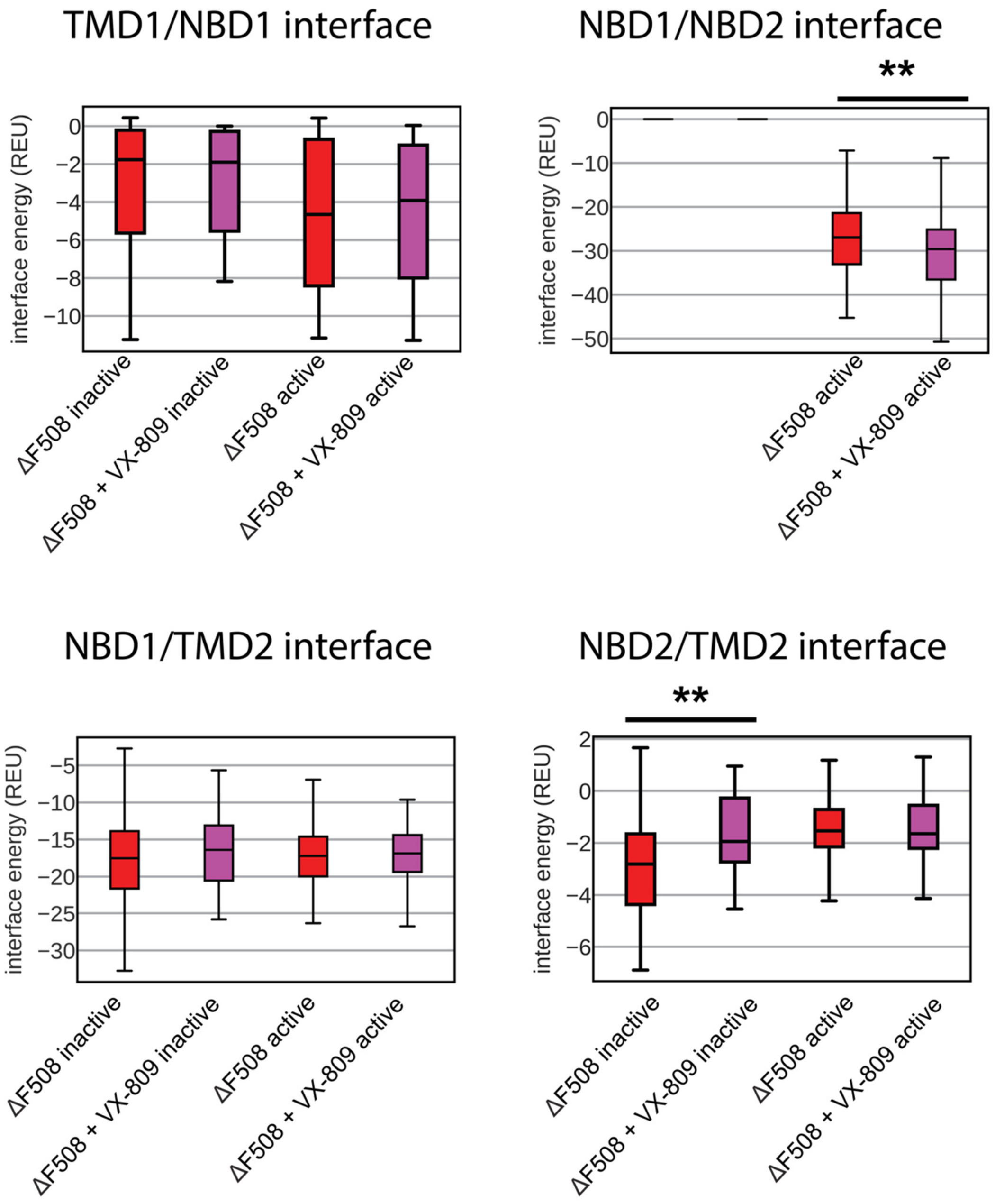

Supplemental Figure S13. F508del + VX-809 interface energy calculations. Quantification of the residue-residue interactions at the domain-domain interface across the lowest scoring 100 models between F508del and F508del + VX-809. (* - p< 0.05, ** - p < 0.01, *** - p < 0.001, **** - p < 0.0001). Statistical significance was calculated with a Mann-Whitney U test.

SUPPLEMENTAL FIGURE S14

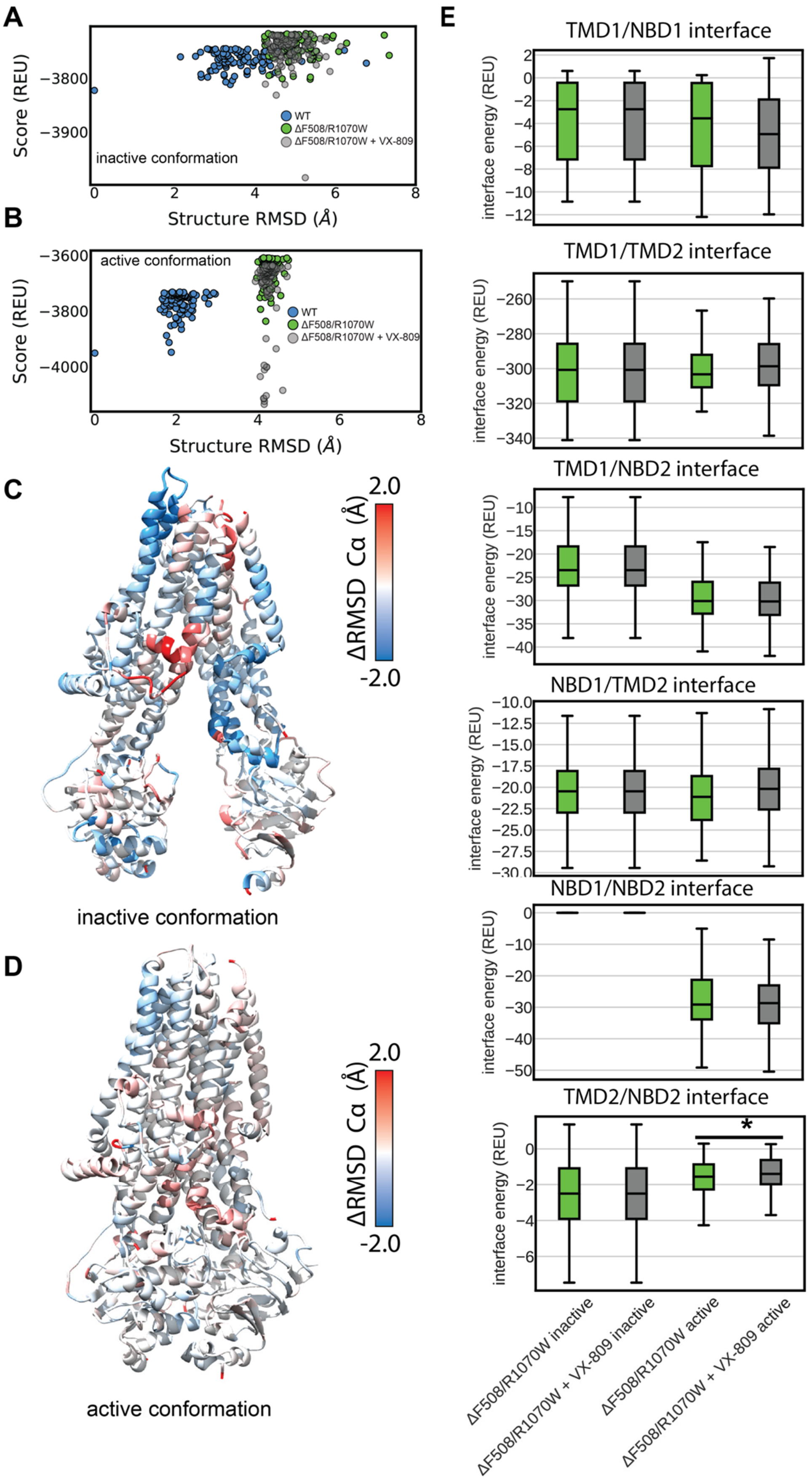

Supplemental Figure S14. F508del/R1070W + VX-809 score versus RMSD plots, ensemble diversity, and interface energy calculations. **A**. Cα RMSD vs. score plot of the lowest scoring 100 inactive conformation models from ensembles of WT (blue) and F508del/R1070W (green), and F508del/R1070W + VX-809 (translucent grey). RMSD is calculated relative to the lowest scoring WT model. Score is shown in REU. **B.** Cα RMSD vs. score plot of the lowest scoring 100 active conformation models from ensembles of WT (blue) and F508del/R1070W (green), and F508del/R1070W + VX-809 (translucent grey). RMSD is calculated relative to the lowest scoring WT model. Score is shown in REU. **C.** Average residue Cα RMSD of the lowest scoring 100 inactive state F508del/R1070W models subtracted from the Cα RMSD of the lowest scoring 100 inactive state F508del/R1070W + VX809 models mapped on 5UAK. Here blue represents regions where VX-809 stabilized F508del/R1070W. **D.** Average residue Cα RMSD of the lowest scoring 100 inactive state F508del/R1070W models subtracted from the Cα RMSD of the lowest scoring 100 inactive state F508del/R1070W + VX-809 models mapped on 6MSM. **E.** Quantification of the residue-residue interactions at each domain-domain interface across the lowest scoring 100 models between F508del/R1070W and F508del/R1070W + VX-809 (* - $p< 0.05$). Statistical significance was calculated with a Mann-Whitney U test. VX-809 does not stabilize any domain-domain interface for F508del/R1070W but may destabilize the TMD2/NBD2 interface.

SUPPLEMENTAL TABLE S1

Structure	Residues with Coordinates	Fasta sequence
5uak	5-402, 439-645, 845-883, 909-1172, 1207-1436	PLEKASVVSKLFFSWTRPILRKGYRQRLELSDIYQIPSVDSADNLSEKLEREWDRELA<br>SKKNPKLINALRRCFFWRFMFYGIFLYLGEVTKAVQPLLLGRIIASYDPDNKEERSIAI<br>YLGIGLCLLFIVRTLLLHPAIFGLHHIGMQMRIAMFSLIYKKTLKLSSRVLDKISIGQL<br>VSLLSNNLNKFDEGLALAHFVWIAPLQVALLMGLIWELLQASAFCGLGFLIVLALFQ<br>AGLGRMMMKYRDQRAGKISERLVITSEMIENIQSVKAYCWEEAMEKMIENLRQTEL<br>KLTRKAAYVRYFNSSAFFFSGFFVVFLSVLPYALIKGIILRKIFTTISFCIVLRMAVTRQ<br>FPWAVQTWYDSLGAINKIQDFLQKQEYKTLEYNLTTTEVVMENVTAFWEPVLKDIN<br>FKIERGQLLAVAGSTGAGKTSLLMVIMGELEPSEGKIKHSGRISFCSQFSWIMPGTIKE<br>NIIFGVSYDEYRYRSVIKACQLEEDISKFAEKDNIVLGEGGITLSGGQRARISLARAVY<br>KDADLYLLDSPFGYLDVLTEKEIFESCVCKLMANKTRILVTSKMEHLKKADKILILH<br>EGSSYFYGTFSELQNLQPDFSSKLMTTWNTYLRYITVHKSLIFVLIWCLVIFLAEVAA<br>SLVVLWLSTSSYYVFYIYVGVADTLLAMGFFRGLPLVHTLITVSKILHHKMLHSVLQ<br>APMSTLNTLKAGGILNRFSKDIAILDDLLPLTIFDFIQLLLIVIGAIAVVAVLQPYIFVA<br>TVPVIVAFIMLRAYFLQTSQQLKQLESEGRSPIFTHLVTSLKGLWTLRAFGRQPYFET<br>LFHKALNLHTANWFLYLSTLRWFQMRIEMIFVIFFIAVTFISILTTGEGEGRVGIILTLA<br>MNIMSTLQWAVNSSIDVDSLMRSVSRVFKFIDMPTEGGQMTVKDLTAKYTEGGNAI<br>LENISFSISPGQRVGLLGRTGSGKSTLLSAFLRLLNTEGEIQIDGVSWDSITLQQWRKA<br>FGVIPQKVFIFSGTFRKNLDPYEQWSDQEIWKVADEVGLRSVIEQFPGKLDFVLVDG<br>GCVLSHGHKQLMCLARSVLSKAKILLLDEPSAHLDPVTYQIIRRTLKQAFADCTVILC<br>EHRIEAMLECQQFLVIEENKVRQYDSIQKLLNERSL
6msm	1-409, 435-637, 845-889, 900-1173, 1202-1451	MQRSPLEKASVVSKLFFSWTRPILRKGYRQRLELSDIYQIPSVDSADNLSEKLEREWD<br>RELASKKNPKLINALRRCFFWRFMFYGIFLYLGEVTKAVQPLLLGRIIASYDPDNKEE<br>RSIAIYLGIGLCLLFIVRTLLLHPAIFGLHHIGMQMRIAMFSLIYKKTLKLSSRVLDKISI<br>GQLVSLLSNNLNKFDEGLALAHFVWIAPLQVALLMGLIWELLQASAFCGLGFLIVLA<br>LFQAGLGRMMMKYRDQRAGKISERLVITSEMIENIQSVKAYCWEEAMEKMIENLRQ<br>TELKLTRKAAYVRYFNSSAFFFSGFFVVFLSVLPYALIKGIILRKIFTTISFCIVLRMAV<br>TRQFPWAVQTWYDSLGAINKIQDFLQKQEYKTLEYNLTTTEVVMENVTAFWEEGF<br>GELFLLGTPVLKDINFKIERGQLLAVAGSTGAGKTSLLMVIMGELEPSEGKIKHSGRI<br>SFCSQFSWIMPGTIKENIIFGVSYDEYRYRSVIKACQLEEDISKFAEKDNIVLGEGGITL<br>SGGQRARISLARAVYKDADLYLLDSPFGYLDVLTEKEIFESCVCKLMANKTRILVTS<br>KMEHLKKADKILILHEGSSYFYGTFSELQNLQTWNTYLRYITVHKSLIFVLIWCLVIF<br>LAEVAASLVVLWLLGNTPLNNSYAVIITSTSSYYVFYIYVGVADTLLAMGFFRGLPL<br>VHTLITVSKILHHKMLHSVLQAPMSTLNTLKAGGILNRFSKDIAILDDLLPLTIFDFIQ

		LLLIVIGAIAVVAVLQPYIFVATVPVIVAFIMLRAYFLQTSQQLKQLESEGRSPIFTHL<br>VTSLKGLWTLRAFGRQPYFETLFHKALNLHTANWFLYLSTLRWFQMRIEMIFVIFFI<br>AVTFISILTTGEGEGRVGIILTLAMNIMSTLQWAVNSSIDVDSLMRSVSRVFKFIDMPT<br>EGDIWPSGGQMTVKDLTAKYTEGGNAILENISFSISPGQRVGLLGRTGSGKSTLLSAF<br>LRLLNTEGEIQIDGVSWDSITLQQWRKAFGVIPQKVFIFSGTFRKNLDPYEQWSDQEI<br>WKVADEVGLRSVIEQFPGKLDFVLVDGGCVLSHGHKQLMCLARSVLSKAKILLLDE<br>PSAHLDPVTYQIIRRTLKQAFADCTVILCEHRIEAMLECQQFLVIEENKVRQYDSIQK<br>LLNERSLFRQAISPSDRVKLFP

Supplemental Table S1. Model Fasta Sequences. PDB ID, determined residues, and respective FASTA sequence of the published cryo-EM structures used in this study.

SUPPLEMENTAL TABLE S2

	Construct	Tm (ºC)	ΔTm (ºC)	Referemce	ΔGº (kcal/mol)	ΔΔGº (kcal/mol)	Reference
ΔRI	WT	57.7	0	Protasevich et al 2010	-5.988	0	Wang et al 2010
	F494N	42	-1.4	Rabeh et al 2012	N/A	-0.5	Rabeh et al 2012
	F494N/Q637R	59.2	-1.5	Protasevich et al 2010	-4.585	-1.403	Wang et al 2010
	V510D	60.2	-2.5	Protasevich et al 2010	-5.987	-0.001	Wang et al 2010
	I539T		-2.5	Mendoza et al. 2012			
	G550E/R553Q/R555K	61.7	-4	Protasevich et al 2010	-7.585	1.597	Wang et al 2011
	F508del	51.5	6.2	Protasevich et al 2010	-3.283	-2.705	Wang et al 2011
	F508del/F494N	34	5.8	Rabeh et al 2012		-2	Rabeh et al 2012
	F508del/F494N/Q637R	52.8	4.9	Protasevich et al 2010	-3.674	-2.314	Wang et al 2011
	F508del/V510D	53	4.7	Protasevich et al 2010	-2.808	-3.18	Wang et al 2011

	F508del/I539T		0.1	Mendoza et al. 2012			
	F508del/G550E/R553Q/R555K	55.7	2	Protasevich et al 2010	-5.143	-0.845	Wang et al 2011

Supplemental Table S2. CFTR NBD1 Tm and ΔG thermodynamic data for testing against RosettaCM template number. Literature values for experimental ΔT_M and ΔΔG data used in testing portion of this study. References shown in table (3–5).

REFERENCES

1. Liu F, Zhang Z, Csanády L, Gadsby DC, Chen J. Molecular Structure of the Human CFTR Ion Channel. Cell. 2017;169(1):85–92.
2. Zhang Z, Liu F, Chen J. Molecular structure of the ATP-bound, phosphorylated human CFTR. Proc Natl Acad Sci. 2018;115(50):12757–62.
3. Protasevich I, Yang Z, Wang C, Atwell S, Zhao X, Emtage S, et al. Thermal unfolding studies show the disease causing F508del mutation in CFTR thermodynamically destabilizes nucleotide-binding domain 1. Protein Sci. 2010;19(10):1917–31.
4. Rabeh WM, Bossard F, Xu H, Okiyoneda T, Bagdany M, Mulvihill CM, et al. Correction of both NBD1 energetics and domain interface is required to restore Δf508 CFTR folding and function. Cell [Internet]. 2012;148(1–2):150–63. Available from: http://dx.doi.org/10.1016/j.cell.2011.11.024
5. Mendoza JL, Schmidt A, Li Q, Nuvaga E, Barrett T, Bridges RJ, et al. Requirements for efficient correction of Δf508 CFTR revealed by analyses of evolved sequences. Cell. 2012;

Protocol Capture

Step 1: Refinement

In this step you will refine a number of structures into the published CFTR cryo-EM density. First, the following files can be downloaded from https://www.rcsb.org/:

- https://www.rcsb.org/structure/5UAK
    - 5uak.pdb
    - emd_8516.map
- https://www.rcsb.org/structure/6MSM
    - 6msm.pdb
    - emd_9230.map

Refinement options and execution for refining 5UAK into cryo-EM density

```
/dors/meilerlab/apps/rosetta/rosetta-3.10/main/source/bin/rosetta_scripts.linuxgc
crelease -in:file:s 5uak.pdb -in:missing_density_to_jump -parser:protocol
A_asymm_refine.xml -nstruct 1 -parser:script_vars denswt=30 rms=1.75 reso=3.87
map=emd_8516.map testmap=emd_8516.map -ignore_unrecognized_res -edensity::mapreso
3.87 -default_max_cycles 200 -edensity::cryoem_scatterers -beta -crystal_refine
```

Refinement options and execution for refining 6MSM into cryo-EM

```
/dors/meilerlab/apps/rosetta/rosetta-3.10/main/source/bin/rosetta_scripts.linuxgc
crelease -in:file:s 6msm_WT.pdb -in:missing_density_to_jump -parser:protocol
A_asymm_refine.xml -nstruct 1 -parser:script_vars denswt=30 rms=1.75 reso=3.20
map=emd_9230.map testmap=emd_9230.map -ignore_unrecognized_res -edensity::mapreso
3.20 -default_max_cycles 200 -edensity::cryoem_scatterers -beta -crystal_refine
```

RosettaScripts file for refinement reproduced from
https://faculty.washington.edu/dimaio/files/rosetta_density_tutorial_aug18.pdf

`A_asymm_refine.xml`

```
<ROSETTASCRIPTS>
   <SCOREFXNS>
      <ScoreFunction name="cen" weights="score4_smooth_cart">
         <Reweight scoretype="elec_dens_fast" weight="20"/>
      </ScoreFunction>
      <ScoreFunction name="dens_soft" weights="beta_soft">
         <Reweight scoretype="cart_bonded" weight="0.5"/>
         <Reweight scoretype="pro_close" weight="0.0"/>
         <Reweight scoretype="elec_dens_fast" weight="%%denswt%%"/>
      </ScoreFunction>
      <ScoreFunction name="dens" weights="beta_cart">
         <Reweight scoretype="elec_dens_fast" weight="%%denswt%%"/>
         <Set
scale_sc_dens_byres="R:0.76,K:0.76,E:0.76,D:0.76,M:0.76,C:0.81,Q:0.81,H:0.81,N:0.
81,T:0.81,S:0.81,Y:0.88,W:0.88,A:0.88,F:0.88,P:0.88,I:0.88,L:0.88,V:0.88"/>
      </ScoreFunction>
   </SCOREFXNS>
```

```
<MOVERS>
      <SetupForDensityScoring name="setupdens"/>
      <LoadDensityMap name="loaddens" mapfile="%%map%%"/>

      <SwitchResidueTypeSetMover name="tocen" set="centroid"/>

      <MinMover name="cenmin" scorefxn="cen" type="lbfgs_armijo_nonmonotone"
         max_iter="200" tolerance="0.00001" bb="1" chi="1" jump="ALL"/>

      <CartesianSampler name="cen5_50" automode_scorecut="-0.5" scorefxn="cen"
         mcscorefxn="cen" fascorefxn="dens_soft" strategy="auto"
fragbias="density"
         rms="%%rms%%" ncycles="200" fullatom="0" bbmove="1" nminsteps="25"
temp="4" fraglens="13"
         nfrags="25"/>
      <CartesianSampler name="cen5_60" automode_scorecut="-0.3" scorefxn="cen"
         mcscorefxn="cen" fascorefxn="dens_soft" strategy="auto"
fragbias="density"
         rms="%%rms%%" ncycles="200" fullatom="0" bbmove="1" nminsteps="25"
temp="4" fraglens="13"
         nfrags="25"/>
      <CartesianSampler name="cen5_70" automode_scorecut="-0.1" scorefxn="cen"
         mcscorefxn="cen" fascorefxn="dens_soft" strategy="auto"
fragbias="density"
         rms="%%rms%%" ncycles="200" fullatom="0" bbmove="1" nminsteps="25"
temp="4" fraglens="13"
         nfrags="25"/>
      <CartesianSampler name="cen5_80" automode_scorecut="0.0" scorefxn="cen"
         mcscorefxn="cen" fascorefxn="dens_soft" strategy="auto"
fragbias="density"
         rms="%%rms%%" ncycles="200" fullatom="0" bbmove="1" nminsteps="25"
temp="4" fraglens="13"
         nfrags="25"/>

      <ReportFSC name="report" testmap="%%testmap%%" res_low="10.0"
res_high="%%reso%%"/>

      <BfactorFitting name="fit_bs" max_iter="50" wt_adp="0.0005" init="1"
exact="1"/>

      <FastRelax name="relaxcart" scorefxn="dens" repeats="1" cartesian="1"/>
   </MOVERS>
<PROTOCOLS>
      <Add mover="setupdens"/>
      <Add mover="loaddens"/>
      <Add mover="tocen"/>
      <Add mover="cenmin"/>
      <Add mover="relaxcart"/>
      <Add mover="cen5_50"/>
      <Add mover="relaxcart"/>
      <Add mover="cen5_60"/>
      <Add mover="relaxcart"/>
      <Add mover="cen5_70"/>
      <Add mover="relaxcart"/>
      <Add mover="cen5_80"/>
      <Add mover="relaxcart"/>
      <Add mover="relaxcart"/>
      <Add mover="report"/>
   </PROTOCOLS>
   <OUTPUT scorefxn="dens"/>
```

Select the lowest scoring 5 models from refinement to use as templates for RosettaCM.

Step 2: Threading F508del Sequence onto templates

For the inactive state, given a template PDB 5uak_###.pdb use the following grishin alignment file.

alignment.grishin

```
## 5uak_F508del.fasta 5uak_####.pdb
#
scores_from_program: 0
0
PLEKASVVSKLFFSWTRPILRKGYRQRLELSDIYQIPSVDSADNLSEKLEREWDRELASKKNPKLINALRRCFFWRFMFYGI
FLYLGEVTKAVQPLLLGRIIASYDPDNKEERSIAIYLGIGLCLLFIVRTLLLHPAIFGLHHIGMQMRIAMFSLIYKKTLKLS
SRVLDKISIGQLVSLLSNNLNKFDEGLALAHFVWIAPLQVALLMGLIWELLQASAFCGLGFLIVLALFQAGLGRMMMKYRDQ
RAGKISERLVITSEMIENIQSVKAYCWEEAMEKMIENLRQTELKLTRKAAYVRYFNSSAFFFSGFFVVFLSVLPYALIKGII
LRKIFTTISFCIVLRMAVTRQFPWAVQTWYDSLGAINKIQDFLQKQEYKTLEYNLTTTEVVMENVTAFWEPVLKDINFKIER
GQLLAVAGSTGAGKTSLLMVIMGELEPSEGKIKHSGRISFCSQFSWIMPGTIKENII-GVSYDEYRYRSVIKACQLEEDISK
FAEKDNIVLGEGGITLSGGQRARISLARAVYKDADLYLLDSPFGYLDVLTEKEIFESCVCKLMANKTRILVTSKMEHLKKAD
KILILHEGSSYFYGTFSELQNLQPDFSSKLMTTWNTYLRYITVHKSLIFVLIWCLVIFLAEVAASLVVLWLSTSSYYVFYIY
VGVADTLLAMGFFRGLPLVHTLITVSKILHHKMLHSVLQAPMSTLNTLKAGGILNRFSKDIAILDDLLPLTIFDFIQLLLIV
IGAIAVVAVLQPYIFVATVPVIVAFIMLRAYFLQTSQQLKQLESEGRSPIFTHLVTSLKGLWTLRAFGRQPYFETLFHKALN
LHTANWFLYLSTLRWFQMRIEMIFVIFFIAVTFISILTTGEGEGRVGIILTLAMNIMSTLQWAVNSSIDVDSLMRSVSRVFK
FIDMPTEGGQMTVKDLTAKYTEGGNAILENISFSISPGQRVGLLGRTGSGKSTLLSAFLRLLNTEGEIQIDGVSWDSITLQQ
WRKAFGVIPQKVFIFSGTFRKNLDPYEQWSDQEIWKVADEVGLRSVIEQFPGKLDFVLVDGGCVLSHGHKQLMCLARSVLSK
AKILLLDEPSAHLDPVTYQIIRRTLKQAFADCTVILCEHRIEAMLECQQFLVIEENKVRQYDSIQKLLNERSL
0
PLEKASVVSKLFFSWTRPILRKGYRQRLELSDIYQIPSVDSADNLSEKLEREWDRELASKKNPKLINALRRCFFWRFMFYGI
FLYLGEVTKAVQPLLLGRIIASYDPDNKEERSIAIYLGIGLCLLFIVRTLLLHPAIFGLHHIGMQMRIAMFSLIYKKTLKLS
SRVLDKISIGQLVSLLSNNLNKFDEGLALAHFVWIAPLQVALLMGLIWELLQASAFCGLGFLIVLALFQAGLGRMMMKYRDQ
RAGKISERLVITSEMIENIQSVKAYCWEEAMEKMIENLRQTELKLTRKAAYVRYFNSSAFFFSGFFVVFLSVLPYALIKGII
LRKIFTTISFCIVLRMAVTRQFPWAVQTWYDSLGAINKIQDFLQKQEYKTLEYNLTTTEVVMENVTAFWEPVLKDINFKIER
GQLLAVAGSTGAGKTSLLMVIMGELEPSEGKIKHSGRISFCSQFSWIMPGTIKENIIFGVSYDEYRYRSVIKACQLEEDISK
FAEKDNIVLGEGGITLSGGQRARISLARAVYKDADLYLLDSPFGYLDVLTEKEIFESCVCKLMANKTRILVTSKMEHLKKAD
KILILHEGSSYFYGTFSELQNLQPDFSSKLMTTWNTYLRYITVHKSLIFVLIWCLVIFLAEVAASLVVLWLSTSSYYVFYIY
VGVADTLLAMGFFRGLPLVHTLITVSKILHHKMLHSVLQAPMSTLNTLKAGGILNRFSKDIAILDDLLPLTIFDFIQLLLIV
IGAIAVVAVLQPYIFVATVPVIVAFIMLRAYFLQTSQQLKQLESEGRSPIFTHLVTSLKGLWTLRAFGRQPYFETLFHKALN
LHTANWFLYLSTLRWFQMRIEMIFVIFFIAVTFISILTTGEGEGRVGIILTLAMNIMSTLQWAVNSSIDVDSLMRSVSRVFK
FIDMPTEGGQMTVKDLTAKYTEGGNAILENISFSISPGQRVGLLGRTGSGKSTLLSAFLRLLNTEGEIQIDGVSWDSITLQQ
WRKAFGVIPQKVFIFSGTFRKNLDPYEQWSDQEIWKVADEVGLRSVIEQFPGKLDFVLVDGGCVLSHGHKQLMCLARSVLSK
AKILLLDEPSAHLDPVTYQIIRRTLKQAFADCTVILCEHRIEAMLECQQFLVIEENKVRQYDSIQKLLNERSL
```

For the inactive state, use the following fasta file for F508del with slashes to indicate breaks in the structure.

5uak_F508del.fasta

```
>5uak_F508del
PLEKASVVSKLFFSWTRPILRKGYRQRLELSDIYQIPSVDSADNLSEKLEREWDRELASKKNPKLINALRRCFFWRFMFYGI
FLYLGEVTKAVQPLLLGRIIASYDPDNKEERSIAIYLGIGLCLLFIVRTLLLHPAIFGLHHIGMQMRIAMFSLIYKKTLKLS
SRVLDKISIGQLVSLLSNNLNKFDEGLALAHFVWIAPLQVALLMGLIWELLQASAFCGLGFLIVLALFQAGLGRMMMKYRDQ
RAGKISERLVITSEMIENIQSVKAYCWEEAMEKMIENLRQTELKLTRKAAYVRYFNSSAFFFSGFFVVFLSVLPYALIKGII
LRKIFTTISFCIVLRMAVTRQFPWAVQTWYDSLGAINKIQDFLQKQEYKTLEYNLTTTEVVMENVTAFWE/PVLKDINFKIE
RGQLLAVAGSTGAGKTSLLMVIMGELEPSEGKIKHSGRISFCSQFSWIMPGTIKENIIGVSYDEYRYRSVIKACQLEEDISK
FAEKDNIVLGEGGITLSGGQRARISLARAVYKDADLYLLDSPFGYLDVLTEKEIFESCVCKLMANKTRILVTSKMEHLKKAD
KILILHEGSSYFYGTFSELQNLQPDFSSKLM/TTWNTYLRYITVHKSLIFVLIWCLVIFLAEVAASLVVLWL/STSSYYVFY
IYVGVADTLLAMGFFRGLPLVHTLITVSKILHHKMLHSVLQAPMSTLNTLKAGGILNRFSKDIAILDDLLPLTIFDFIQLLL
IVIGAIAVVAVLQPYIFVATVPVIVAFIMLRAYFLQTSQQLKQLESEGRSPIFTHLVTSLKGLWTLRAFGRQPYFETLFHKA
LNLHTANWFLYLSTLRWFQMRIEMIFVIFFIAVTFISILTTGEGEGRVGIILTLAMNIMSTLQWAVNSSIDVDSLMRSVSRV
FKFIDMPTE/GGQMTVKDLTAKYTEGGNAILENISFSISPGQRVGLLGRTGSGKSTLLSAFLRLLNTEGEIQIDGVSWDSIT
LQQWRKAFGVIPQKVFIFSGTFRKNLDPYEQWSDQEIWKVADEVGLRSVIEQFPGKLDFVLVDGGCVLSHGHKQLMCLARSV
LSKAKILLLDEPSAHLDPVTYQIIRRTLKQAFADCTVILCEHRIEAMLECQQFLVIEENKVRQYDSIQKLLNERSL
```

Execute threading onto inactive state templates using the following command.

```
/dors/meilerlab/apps/rosetta/rosetta-
3.11/main/source/bin/partial_thread.linuxgccrelease -in:file:fasta
5uak_F508del.fasta -in:file:alignment alignment.grishin -in:file:template_pdb
5uak_####.pdb -ignore_unrecognized_res
```

For the active state, given a template PDB 6msm_WT_###.pdb use the following grishin alignment file. Note, that WT designation is given because the published model contains an E1371Q stabilizing mutation which must be mutated using the MutateResidueMover in Rosetta prior to threading and RosettaCM.

alignment.grishin

```
## 6msm_F508del.fasta 6msm_WT_####.pdb
#
scores_from_program: 0
0
MQRSPLEKASVVSKLFFSWTRPILRKGYRQRLELSDIYQIPSVDSADNLSEKLEREWDRELASKKNPKLINALRRCFFWRF
MFYGIFLYLGEVTKAVQPLLLGRIIASYDPDNKEERSIAIYLGIGLCLLFIVRTLLLHPAIFGLHHIGMQMRIAMFSLIYK
KTLKLSSRVLDKISIGQLVSLLSNNLNKFDEGLALAHFVWIAPLQVALLMGLIWELLQASAFCGLGFLIVLALFQAGLGRM
MMKYRDQRAGKISERLVITSEMIENIQSVKAYCWEEAMEKMIENLRQTELKLTRKAAYVRYFNSSAFFFSGFFVVFLSVLP
YALIKGIILRKIFTTISFCIVLRMAVTRQFPWAVQTWYDSLGAINKIQDFLQKQEYKTLEYNLTTTEVVMENVTAFWEEGF
GELFLLGTPVLKDINFKIERGQLLAVAGSTGAGKTSLLMVIMGELEPSEGKIKHSGRISFCSQFSWIMPGTIKENII-GVS
YDEYRYRSVIKACQLEEDISKFAEKDNIVLGEGGITLSGGQRARISLARAVYKDADLYLLDSPFGYLDVLTEKEIFESCVC
KLMANKTRILVTSKMEHLKKADKILILHEGSSYFYGTFSELQNLQTWNTYLRYITVHKSLIFVLIWCLVIFLAEVAASLVV
LWLLGNTPLNNSYAVIITSTSSYYVFYIYVGVADTLLAMGFFRGLPLVHTLITVSKILHHKMLHSVLQAPMSTLNTLKAGG
ILNRFSKDIAILDDLLPLTIFDFIQLLLIVIGAIAVVAVLQPYIFVATVPVIVAFIMLRAYFLQTSQQLKQLESEGRSPIF
THLVTSLKGLWTLRAFGRQPYFETLFHKALNLHTANWFLYLSTLRWFQMRIEMIFVIFFIAVTFISILTTGEGEGRVGIIL
TLAMNIMSTLQWAVNSSIDVDSLMRSVSRVFKFIDMPTEGDIWPSGGQMTVKDLTAKYTEGGNAILENISFSISPGQRVGL
LGRTGSGKSTLLSAFLRLLNTEGEIQIDGVSWDSITLQQWRKAFGVIPQKVFIFSGTFRKNLDPYEQWSDQEIWKVADEVG
LRSVIEQFPGKLDFVLVDGGCVLSHGHKQLMCLARSVLSKAKILLLDEPSAHLDPVTYQIIRRTLKQAFADCTVILCEHRI
EAMLECQQFLVIEENKVRQYDSIQKLLNERSLFRQAISPSDRVKLFP
0
MQRSPLEKASVVSKLFFSWTRPILRKGYRQRLELSDIYQIPSVDSADNLSEKLEREWDRELASKKNPKLINALRRCFFWRF
MFYGIFLYLGEVTKAVQPLLLGRIIASYDPDNKEERSIAIYLGIGLCLLFIVRTLLLHPAIFGLHHIGMQMRIAMFSLIYK
KTLKLSSRVLDKISIGQLVSLLSNNLNKFDEGLALAHFVWIAPLQVALLMGLIWELLQASAFCGLGFLIVLALFQAGLGRM
MMKYRDQRAGKISERLVITSEMIENIQSVKAYCWEEAMEKMIENLRQTELKLTRKAAYVRYFNSSAFFFSGFFVVFLSVLP
YALIKGIILRKIFTTISFCIVLRMAVTRQFPWAVQTWYDSLGAINKIQDFLQKQEYKTLEYNLTTTEVVMENVTAFWEEGF
GELFLLGTPVLKDINFKIERGQLLAVAGSTGAGKTSLLMVIMGELEPSEGKIKHSGRISFCSQFSWIMPGTIKENIIFGVS
YDEYRYRSVIKACQLEEDISKFAEKDNIVLGEGGITLSGGQRARISLARAVYKDADLYLLDSPFGYLDVLTEKEIFESCVC
KLMANKTRILVTSKMEHLKKADKILILHEGSSYFYGTFSELQNLQTWNTYLRYITVHKSLIFVLIWCLVIFLAEVAASLVV
LWLLGNTPLNNSYAVIITSTSSYYVFYIYVGVADTLLAMGFFRGLPLVHTLITVSKILHHKMLHSVLQAPMSTLNTLKAGG
ILNRFSKDIAILDDLLPLTIFDFIQLLLIVIGAIAVVAVLQPYIFVATVPVIVAFIMLRAYFLQTSQQLKQLESEGRSPIF
THLVTSLKGLWTLRAFGRQPYFETLFHKALNLHTANWFLYLSTLRWFQMRIEMIFVIFFIAVTFISILTTGEGEGRVGIIL
TLAMNIMSTLQWAVNSSIDVDSLMRSVSRVFKFIDMPTEGDIWPSGGQMTVKDLTAKYTEGGNAILENISFSISPGQRVGL
LGRTGSGKSTLLSAFLRLLNTEGEIQIDGVSWDSITLQQWRKAFGVIPQKVFIFSGTFRKNLDPYEQWSDQEIWKVADEVG
LRSVIEQFPGKLDFVLVDGGCVLSHGHKQLMCLARSVLSKAKILLLDEPSAHLDPVTYQIIRRTLKQAFADCTVILCEHRI
EAMLECQQFLVIEENKVRQYDSIQKLLNERSLFRQAISPSDRVKLFP
```

For the active state, use the following fasta file for F508del with slashes to indicate breaks in the structure.

6msm_F508del.fasta

```
>6msm_F508del.fasta
MQRSPLEKASVVSKLFFSWTRPILRKGYRQRLELSDIYQIPSVDSADNLSEKLEREWDRELASKKNPKLINALRRCFFWRF
MFYGIFLYLGEVTKAVQPLLLGRIIASYDPDNKEERSIAIYLGIGLCLLFIVRTLLLHPAIFGLHHIGMQMRIAMFSLIYK
KTLKLSSRVLDKISIGQLVSLLSNNLNKFDEGLALAHFVWIAPLQVALLMGLIWELLQASAFCGLGFLIVLALFQAGLGRM
MMKYRDQRAGKISERLVITSEMIENIQSVKAYCWEEAMEKMIENLRQTELKLTRKAAYVRYFNSSAFFFSGFFVVFLSVLP
YALIKGIILRKIFTTISFCIVLRMAVTRQFPWAVQTWYDSLGAINKIQDFLQKQEYKTLEYNLTTTEVVMENVTAFWEEGF
GELF/LLGTPVLKDINFKIERGQLLAVAGSTGAGKTSLLMVIMGELEPSEGKIKHSGRISFCSQFSWIMPGTIKENIIGVS
YDEYRYRSVIKACQLEEDISKFAEKDNIVLGEGGITLSGGQRARISLARAVYKDADLYLLDSPFGYLDVLTEKEIFESCVC
KLMANKTRILVTSKMEHLKKADKILILHEGSSYFYGTFSELQNLQ/TWNTYLRYITVHKSLIFVLIWCLVIFLAEVAASLV
VLWLLGNTPL/NNSYAVIITSTSSYYVFYIYVGVADTLLAMGFFRGLPLVHTLITVSKILHHKMLHSVLQAPMSTLNTLKA
GGILNRFSKDIAILDDLLPLTIFDFIQLLLIVIGAIAVVAVLQPYIFVATVPVIVAFIMLRAYFLQTSQQLKQLESEGRSP
IFTHLVTSLKGLWTLRAFGRQPYFETLFHKALNLHTANWFLYLSTLRWFQMRIEMIFVIFFIAVTFISILTTGEGEGRVGI
ILTLAMNIMSTLQWAVNSSIDVDSLMRSVSRVFKFIDMPTEG/DIWPSGGQMTVKDLTAKYTEGGNAILENISFSISPGQR
VGLLGRTGSGKSTLLSAFLRLLNTEGEIQIDGVSWDSITLQQWRKAFGVIPQKVFIFSGTFRKNLDPYEQWSDQEIWKVAD
EVGLRSVIEQFPGKLDFVLVDGGCVLSHGHKQLMCLARSVLSKAKILLLDEPSAHLDPVTYQIIRRTLKQAFADCTVILCE
HRIEAMLECQQFLVIEENKVRQYDSIQKLLNERSLFRQAISPSDRVKLFP
```

Execute threading onto active state templates using the following command.

```
/dors/meilerlab/apps/rosetta/rosetta-
3.11/main/source/bin/partial_thread.linuxgccrelease -in:file:fasta
5uak_F508del.fasta -in:file:alignment alignment.grishin -in:file:template_pdb
6msm_WT_####.pdb -ignore_unrecognized_res
```

Step 3: CFTR Comparative Modeling

Use the following RosettaScripts file for RosettaCM was used for CFTR comparative modeling.

`rosetta_cm_nocst.xml`

```
<ROSETTASCRIPTS>
    <TASKOPERATIONS>
    </TASKOPERATIONS>
    <SCOREFXNS>
        <ScoreFunction name="stage1"
weights="../../membrane_weights/stage1_membrane.wts" symmetric="0">
            <Reweight scoretype="atom_pair_constraint" weight="0.5"/>
        </ScoreFunction>
        <ScoreFunction name="stage2"
weights="../../membrane_weights/stage2_membrane.wts" symmetric="0">
            <Reweight scoretype="atom_pair_constraint" weight="0.5"/>
        </ScoreFunction>
        <ScoreFunction name="fullatom"
weights="../../membrane_weights/stage3_rlx_membrane.wts" symmetric="0">
            <Reweight scoretype="atom_pair_constraint" weight="0.5"/>
        </ScoreFunction>
        <ScoreFunction name="membrane" weights="membrane_highres_Menv_smooth"
symmetric="0">
            <Reweight scoretype="cart_bonded" weight="0.5"/>
            <Reweight scoretype="pro_close" weight="0"/>
        </ScoreFunction>
        <ScoreFunction name="ref2015" weights="ref2015"/>
    </SCOREFXNS>
<FILTERS>
    </FILTERS>
    <TASKOPERATIONS>
        <InitializeFromCommandline name="commandline_init"/>
        <RestrictToRepacking name="restrict_to_repacking"/>
    </TASKOPERATIONS>
    <MOVERS>
        <Hybridize name="hybridize" stage1_scorefxn="stage1" stage2_scorefxn="stage2"
fa_scorefxn="fullatom" batch="1" stage1_increase_cycles="1.0"
stage2_increase_cycles="1.0" linmin_only="1" frag_1mer_insertion_weight="1.0"
small_frag_insertion_weight="1.0" big_frag_insertion_weight="1.0"
chunk_insertion_weight="1.0" frag_weight_aligned="1.0" max_contig_insertion="10"
add_hetatm="1" >
            <Template pdb="%%template1%%" cst_file="NONE" weight="1.000" />
            <Template pdb="%%template2%%" cst_file="NONE" weight="1.000" />
            <Template pdb="%%template3%%" cst_file="NONE" weight="1.000" />
            <Template pdb="%%template4%%" cst_file="NONE" weight="1.000" />
            <Template pdb="%%template5%%" cst_file="NONE" weight="1.000" />
            <Fragments three_mers="%%3mers%%" nine_mers="%%9mers%%"/>
            <DetailedControls start_res="1" stop_res="%%stop2%%" sample_template="1"
sample_abinitio="0"/>
        </Hybridize>
        <ClearConstraintsMover name="clearconstraints"/>
<FastRelax name="relax" scorefxn="membrane" repeats="1" dualspace="0" bondangle="1"/>
    </MOVERS>
    <APPLY_TO_POSE>
    </APPLY_TO_POSE>
    <PROTOCOLS>
        <Add mover="hybridize"/>
        <Add mover="relax"/>
        <Add mover="clearconstraints"/>
    </PROTOCOLS>
    <OUTPUT scorefxn="membrane"/>
```

Note you can repurpose the fasta files from Step 2 for RosettaCM.

Next, use the following membrane stage weights for the Hybridize mover.

stage1_membrane.wts

```
# stage1 weights for hybridization: membrane score weights added
Menv 2.019
Mpair 1.0
Mcbeta 2.5
cenpack 1.0
hs_pair 1.0
ss_pair 1.0
rsigma  1.0
sheet   1.0
vdw     3.0
rg      .1
rama    0.15
linear_chainbreak    2.0
atom_pair_constraint 1.0
Menv_non_helix 2.019
Menv_termini 2.019
Menv_tm_proj 2.019
Mlipo 1.0

STRAND_STRAND_WEIGHTS 0 6
```

stage2_membrane.wts

```
# stage2 weights for hybridization: membrane scoring weights added
hbond_sr_bb 1.17
hbond_lr_bb 1.17
rama        0.15
omega       0.2
rg          0.1
vdw         3.0
Menv 2.019
Mpair 1.0
Mcbeta 2.5
cenpack_smooth  1.0
cart_bonded     0.05
atom_pair_constraint 0.5
Mlipo 1.0
rsigma 1.0
sheet 1.0
ss_pair 1.0
hs_pair 1.0
Menv_non_helix 2.019
Menv_termini 2.019
Menv_tm_proj 2.019
```

stage3_rlx_membrane.wts

```
# stage3 fullatom weights for hybridization: adopted with membrane scores.
METHOD_WEIGHTS ref  0.16 1.7 -0.67 -0.81 0.63 -0.17 0.56 0.24 -0.65 -0.1 -0.34 -0.89
0.02 -0.97 -0.98 -0.37 -0.27 0.29 0.91 0.51
fa_atr  0.8
fa_rep  0.44
fa_sol  0.00
fa_intra_rep 0.004
fa_pair 0.49
fa_dun  0.56
ref     1
hbond_lr_bb 1.17
hbond_sr_bb 1.17
hbond_bb_sc 2.34
hbond_sc 2.2
p_aa_pp     0.32
dslf_ss_dst 0.5
dslf_cs_ang 2
dslf_ss_dih 5
dslf_ca_dih 5
pro_close   1.0
rama    0.2
omega   0.5
atom_pair_constraint    0.5
coordinate_constraint   0.0
cart_bonded     0.5
fa_mbenv 0.3
fa_mbsolv 0.35
Menv_smooth 0.5
```

The following options were flagged.

hybridize.options

```
-in:file:fasta 6msm_F508del.fasta
-in:file:spanfile 6msm_F508del.span
-parser:script_vars template1=6msm_F508del_689.pdb
template2=6msm_F508del_1686.pdb template3=6msm_F508del_712.pdb
template4=6msm_F508del_1721.pdb template5=6msm_F508del_237.pdb
3mers=6msm_F508del.200.3mers 9mers=6msm_F508del.200.9mers stop1=467 start2=487
stop2=1180
-parser:protocol rosetta_cm_nocst.xml
-nstruct 1
-ignore_unrecognized_res
-extra_res_cen ../files/ATP.cen.params
-extra_res_fa  ../files/ATP.fa.params
-extra_improper_file ../files/ATP.fa.tors
-relax:minimize_bond_angles
-relax:minimize_bond_lengths
-relax:jump_move true
-default_max_cycles 200
-relax:min_type lbfgs_armijo_nonmonotone
-relax:jump_move true
-use_bicubic_interpolation
-hybridize:stage1_probability 1.0
-chemical:exclude_patches LowerDNA  UpperDNA Cterm_amidation SpecialRotamer
VirtualBB ShoveBB VirtualDNAPhosphate VirtualNTerm CTermConnect sc_orbitals
pro_hydroxylated_case1 pro_hydroxylated_case2 ser_phosphorylated
thr_phosphorylated  tyr_phosphorylated tyr_sulfated lys_dimethylated
lys_monomethylated  lys_trimethylated lys_acetylated glu_carboxylated
cys_acetylated tyr_diiodinated N_acetylated C_methylamidated
MethylatedProteinCterm
```

Execute RosettaCM templates using the following command.

```
/dors/meilerlab/apps/rosetta/rosetta-3.10/main/source/bin/rosetta_scripts.linuxgc
crelease @hybridize.options
```
